# Advancements in nasal drug delivery system of natural products

**DOI:** 10.3389/fphar.2025.1667517

**Published:** 2025-09-10

**Authors:** Yenna Hsu, Jiajing Yang, Miaoyang Cao, Ting Xu, Jia He, Huarong Hong, Luyun Jiang, Shunlin Peng, Peizheng Xiong

**Affiliations:** ^1^ Chengdu University of Traditional Chinese Medicine, Chengdu, China; ^2^ Hospital of Chengdu University of Traditional Chinese Medicine, Chengdu, China

**Keywords:** nasal administration, natural products, allergic rhinitis, Alzheimer’s disease, nasal drug delivery system

## Abstract

**Background:**

Nasal drug delivery offers a non-invasive route with rapid absorption and the ability to bypass first-pass metabolism, making it promising for central nervous system (CNS) disorders, nasal diseases such as allergic rhinitis, and other chronic conditions by enabling targeted delivery and crossing the blood-brain barrier.

**Purpose:**

To review the advantages of nasal delivery, therapeutic potential of natural products, and how drug delivery systems may overcome bioavailability and solubility issues.

**Study Design:**

A literature review analyzing mechanisms, clinical applications, and limitations of natural products in nasal delivery.

**Methods:**

Relevant articles published before January 2025 were retrieved from Google Scholar, PubMed, ScienceDirect, Scopus, Web of Science, Springer, and official sources.

**Results:**

Nasal administration improves the bioavailability and absorption of natural products, enhancing anti-inflammatory, antioxidant, neuroprotective, and anti-allergic effects. However, poor solubility and stability remain barriers, which may be mitigated by nanocarriers, liposomes, and other advanced systems.

**Conclusion:**

Combining nasal drug delivery with natural products is a promising strategy for treating CNS, nasal, and chronic diseases, potentially improving clinical efficacy and expanding therapeutic options.

## 1 Introduction

Nasal drug delivery has increasingly garnered significant attention in recent years as a non-invasive drug delivery route. Traditional drug delivery methods, such as oral and injectable routes, despite their widespread use in clinical practice, are often constrained by challenges including the rate of drug absorption, duration of efficacy, and associated side effects. Oral drug administration is influenced by digestion and absorption processes in the gastrointestinal tract, while some drugs undergo hepatic first-pass metabolism, leading to diminished therapeutic efficacy. Although injectable drug delivery bypasses the gastrointestinal tract, it remains associated with pain, infection risks, and reduced patient compliance. In contrast, the nasal cavity, serving as a direct route to the brain and systemic circulation, facilitates rapid and efficient drug absorption and is particularly suitable for the treatment of neurological and respiratory diseases, among others (Shrewsbury, 2023) recent years, driven by advancements in drug formulation technologies, nasal drug delivery is increasingly recognised as a promising drug delivery route, demonstrating significant advantages across various clinical domains.

The advantages of nasal drug delivery stem primarily from its unique anatomical and physiological characteristics, including a rich vascular network and high permeability. The nasal cavity offers an extensive surface area and a rich vascular network, facilitating the rapid absorption of active pharmaceutical ingredients. Additionally, its mild pH and reduced enzymatic degradation contribute to its suitability as an ideal route for drug delivery (Laffleur and Bauer, 2021; Teng et al., 2022) pass effects refer to the initial metabolic processing of drugs in the intestinal wall or liver following oral administration, which significantly reduce the drug’s bioavailability, thereby diminishing its therapeutic efficacy. In contrast, direct nasal drug delivery effectively bypasses this metabolic pathway. The extensive vascular network in the nasal cavity facilitates the rapid absorption of drugs into the bloodstream via the nasal mucosa, bypassing hepatic metabolism. This approach avoids the first-pass effect in the gastrointestinal tract and liver, with the potential to markedly enhance the bioavailability of specific compounds. This method effectively avoids the first-pass effect and ensures that the drug can exert its therapeutic effect more efficiently and increase its bioavailability (Anselmo et al., 2019; Lobaina Mato, 2019) addition, nasal administration bypasses invasive procedures like transcutaneous or trans-tissue punctures and is widely regarded as a safer and more convenient alternative. The convenience of nasal medication delivery renders it particularly suitable for patients requiring frequent and long-term medication regimens, including conditions like allergic rhinitis and acute sinusitis. This approach is widely accepted due to its simplicity, enabling patients to self-administer without the need for professional assistance (Teng et al., 2022) administration can produce rapid therapeutic effects through direct action on the localised nasal mucosa or the central nervous system. In the treatment of allergic rhinitis and other upper respiratory diseases, nasal drug administration can target the affected site directly, minimise systemic side effects, and offer improved symptomatic relief (Sun et al., 2019), intranasal administration allows drugs to be absorbed and transported to the brain, a process mediated by two primary pathways. First, drugs may enter the systemic circulation via absorption through the respiratory epithelium and subsequently cross the blood-brain barrier (BBB) to reach the brain. Secondly, drugs can bypass the BBB through the trigeminal and olfactory pathways, directly connecting the nasal cavity to the brain. This mechanism offers a non-invasive alternative for drug delivery. For instance, in the treatment of neurodegenerative diseases like Alzheimer’s disease, nasal drug delivery is regarded as a promising solution to overcome traditional therapeutic bottlenecks (Kashyap and Shukla, 2019; Rajput et al., 2022; Ruan et al., 2024).

Meanwhile, the application of natural products in nasal drug delivery has gained increasing emphasis as one of the hotspots in drug development, since natural products generally have fewer side effects and better biocompatibility compared to chemically synthesized drugs. Many natural products exhibit diverse biological activities, including but not limited to anti-inflammatory, antioxidant, and immunomodulatory effects, making them valuable therapeutic agents for the treatment of respiratory diseases, allergic rhinitis, and neurodegenerative disorders. For example, several plant extracts, including flavonoids and terpenoids, have been shown to possess potent anti-inflammatory effects by reducing nasal inflammation and alleviating symptoms of nasal congestion. Furthermore, the use of natural products for nasal delivery extends beyond localized therapy, allowing for systemic effects through rapid absorption. This is particularly evident in the treatment of brain diseases, where specialized delivery systems enable natural products to directly target the central nervous system via the nasal cavity, offering significant potential as a non-invasive treatment for such conditions.

**FIGURE 1:**
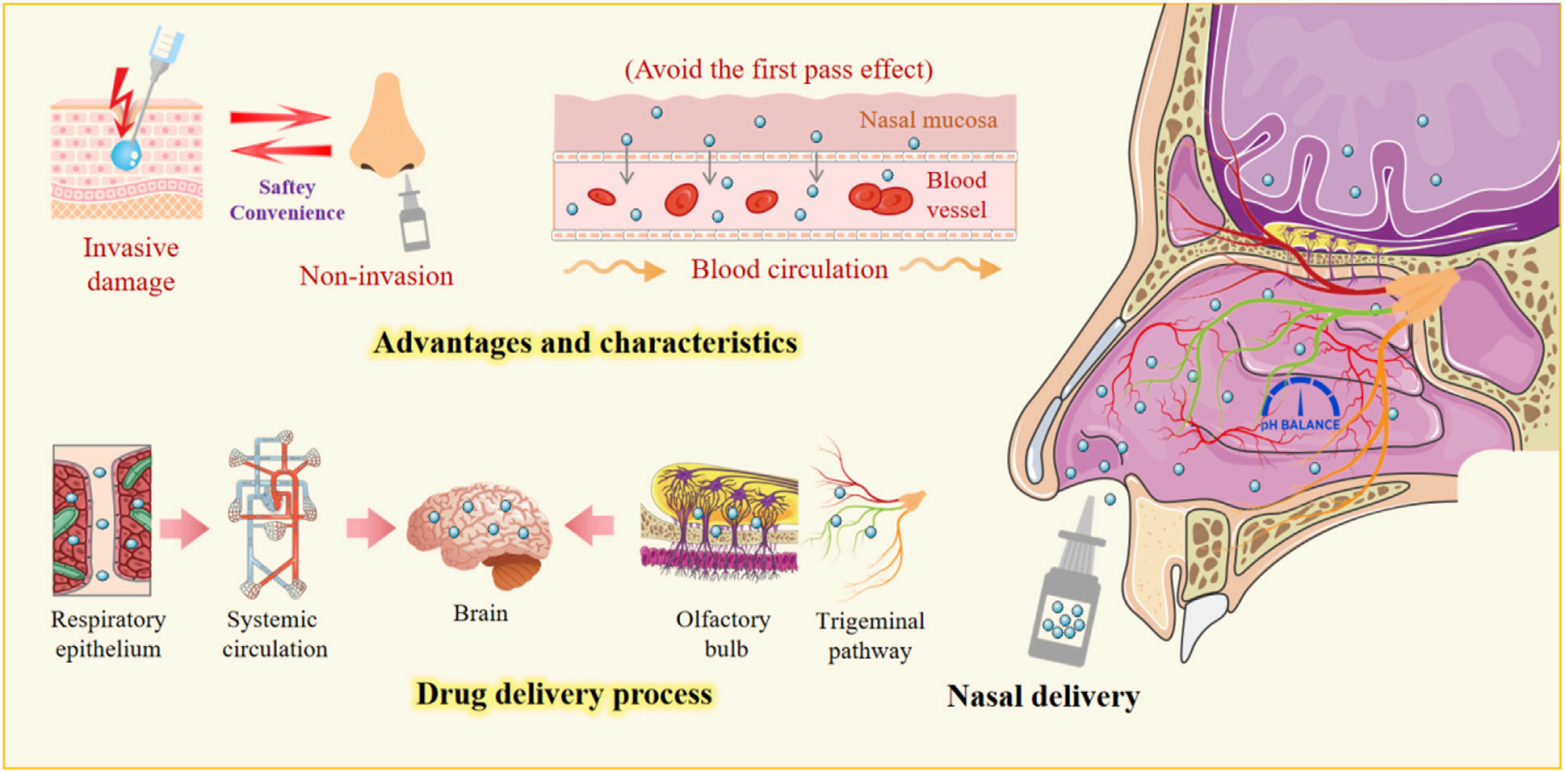
Nasal drug delivery system. This figure highlights the advantages of nasal delivery, emphasizing its non-invasive nature and bypassing the first-pass effect. It shows how drugs pass through the respiratory epithelium into the bloodstream, reaching the brain via the olfactory or trigeminal pathway, offering a safe and effective alternative for CNS targeting.

In contrast to other articles that focus solely on the route of nasal drug delivery and its associated mechanisms of action, this article aims to summarize the latest advances in natural product research. It emphasizes the role of natural products in nasal drug delivery, highlights their advantages when integrated with nasal delivery systems, and explores their therapeutic potential. Furthermore, this paper discusses the technical challenges associated with using natural products in nasal drug delivery for disease treatment and suggests future directions to address these challenges (Figure 1).

## 2 Natural products

Natural products, such as flavonoids, alkaloids, polyphenols, glycosides, and phenylpropanoids, exhibit a wide range of biological activities. The following section will highlight the effects of some natural products and their potential in disease treatment. These specific compound classes were selected based on several core criteria, primarily their extensive research background and representative nature, with substantial literature supporting their potent anti-inflammatory, antioxidant, neuroprotective, and anti-allergic activities. Most importantly, their potential application in treating central nervous system disorders and allergic rhinitis, particularly via the nasal route, has become a major focus of current research with a significant body of evidence. Furthermore, the selected classes cover a variety of chemical structures and mechanisms of action, allowing for a more comprehensive demonstration of their therapeutic potential through nasal delivery systems.

### 2.1 Flavonoids

Flavonoids are a class of polyphenolic compounds characterized by a C6-C3-C6 backbone and are commonly found in plant secondary metabolites. They are abundant in fruits, vegetables, tea, cereals, and various Chinese herbs, and exhibit biological activities including anti-cancer, anti-tumor, anti-cardiovascular, anti-inflammatory, analgesic, antioxidant, immunomodulatory, and bacteriostatic effects (Table 1). While flavonoids show great potential for CNS and allergic diseases, nasal drug delivery systems are crucial for overcoming their poor bioavailability.

**TABLE 1:** Compounds Structure, Bioactivities, and Molecular mechanism of Flavonoids.

Types	Name	Compounds structure	Bioactivities	Molecular mechanism	Reference
Flavonoids	Baicalin	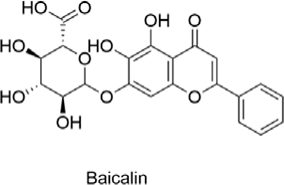	Antiallergic Antioxidant Anti-inflammatory Neuroprotective	1. NF-κB↓, MMP-9↓, IL-1β↓, IL-6↓ 2. IgE↓, Histamine↓, IL-4↓, TNF-α↓	Long et al. (2020), Xiang et al. (2020), Chen et al. (2019)
	Baicalein	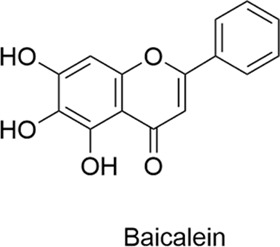	Antiallergic Antioxidant Anti-inflammatory Neuroprotective	Th1/Th2, Balance↑, Histamine↓	Bui et al. (2017)
	Naringenin	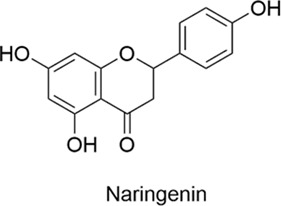	Antiallergic Antioxidant Anti-inflammatory Neuroprotective Antitumor	1. MAO-A↓→ Serotonin↑, Epinephrine↑, Dopamine↑ 2. IgE↓, IL-4↓, IL-5↓ 3. ROS↑→ AMPK↑→ Autophagy↑	Qizilbash et al. (2022), Şahin et al. (2021), Chang et al. (2024)
	Quercetin	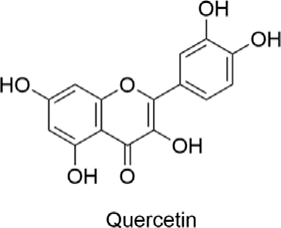	Antiallergic Antioxidant Anti-inflammatory Neuroprotective	1. p-Akt↑, p-ASK1↓, p-JNK3↓, Cleaved Caspase-3↓ → Neuronal Apoptosis↓ 2. mTOR Pathway Modulation → Inflammation↓, Apoptosis↓, Oxidative Stress↓ 3. Th1/Th2 Balance Stabilization → IgE↓, Inflammation↓	Pei et al. (2016), Wang et al. (2022), Jafarinia et al. (2020)
	Hesperidin	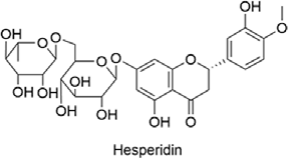	Antiallergic Antioxidant Anti-inflammatory Neuroprotective Cardioprotective	1. RAGE↓, NF-κB↓, Akt/Nrf2↑→ Inflammation↓, Oxidative Stress↓ 2. Nrf2↑, HO-1↑→ Apoptosis↓, ROS↓, MDA↓, SOD↑, GSH↑ 3. IgE↓, IL-5↓, IL-13↓ → Allergic Responses↓	Hong and An (2018), Muhammad et al. (2019), Kilic et al. (2019)
	Kaempferol	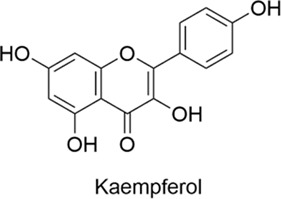	Antiallergic Antioxidant Anti-inflammatory Neuroprotective Cardioprotective Antitumor	1. Apoptosis-related Proteins Regulation → Neuronal Protection 2. Pro-inflammatory Factors↓, mRNA Expression↓ → Inflammation↓ 3. IL-32↓, TSLP↓, Caspase-1 Activity↓	Dong et al. (2023), Oh et al. (2013)
	Luteolin	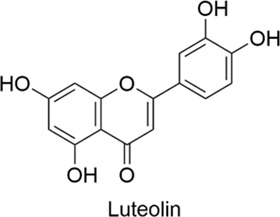	Antiallergic Antioxidant Anti-inflammatory Neuroprotective Cardioprotective Antitumor	1. IgE↓, IgG↓, IL-4↓ → Pro-inflammatory Cytokines↓ 2. TLR4/NF-κB Modulation → Inflammation↓, Th1/Th2 Balance↑	Liang et al. (2020), Dong et al. (2021)
	Mangiferin	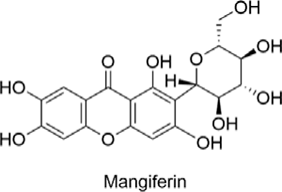	Antiallergic Antioxidant Anti-inflammatory Neuroprotective Cardioprotective	1. Th1/Th2 Balance↑, NF-κB↓, Nrf2/HO-1↑ → Antioxidation↑, Inflammation↓ 2. ATP Depletion↓, Cytochrome c Release↓, Caspase Activation↓→ Neuronal Death↓	Liu et al. (2021), Walia et al. (2021)
	Apigenin	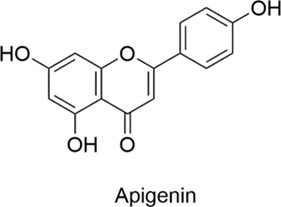	Antiallergic Antioxidant Anti-inflammatory Neuroprotective Cardioprotective	1. TLR4/MyD88/NF-κB↓→ Inflammation↓ 2. Th1↑→ Th1/Th2 Balance↑	Li et al. (2023a), Chen et al. (2020a)

#### 2.1.1 Baicalin

Baicalin (BA), a flavonoid derived from the rhizome of Scutellaria baicalensis, exhibits antioxidant, anti-apoptotic, and anti-inflammatory properties and has demonstrated therapeutic potential in the treatment of brain injury. Specifically, BA inhibits NF-κB activation, downregulates MMP-9 expression, reduces the production of IL-1β and IL-6, and effectively reduces the permeability of the BBB (Long et al., 2020) have shown that the limitations of baicalin, including its poor water and fat solubility, can be overcome through the preparation of baicalin liposomes (BA-LP). Administered via the nose-to-brain drug delivery (NBDD) pathway, BA-LP demonstrates significant brain targeting and enhanced bioavailability, thereby improving drug efficacy and offering a novel therapeutic strategy for ischemic stroke (Xiang et al., 2020) has been shown to mitigate allergic rhinitis (AR) symptoms in rat models by inhibiting the release of immunoglobulin E (IgE), histamine, interleukin-1β, interleukin-4 (IL-4), interleukin-6 (IL-6), and tumor necrosis factor-α (TNF-α) (Chen et al., 2019).

#### 2.1.2 Baicalein

Baicalein and baicalin are flavonoids extracted from the rhizome of the traditional Chinese medicine Scutellaria baicalensis, with distinct differences in chemical structure and biological activity. Both compounds possess anti-inflammatory, antioxidant, and neuroprotective properties. Research suggests that baicalein has the potential to alleviate ovalbumin (OVA)-induced allergic airway inflammation and mast cell-mediated anaphylaxis by modulating the Th1/Th2 balance and inhibiting histamine release (Bui et al., 2017) anti-inflammatory and antioxidant effects of baicalein contribute to its neuroprotective function, which is crucial for the treatment of neurodegenerative diseases of the central nervous system, such as Alzheimer’s and Parkinson’s diseases. These diseases are associated with oxidative stress and inflammation in the brain, but the therapeutic use of baicalein is hampered by its limited solubility and low bioavailability. One of the main challenges is to deliver a sufficient therapeutic dose to the brain. To address this, some studies have encapsulated baicalein in PEG-PLA micelles, which protect the compound during intranasal administration, bypass the first-pass effect, cross the BBB, enhance bioavailability, and improve brain distribution (Zhang et al., 2020a) findings suggest that this approach could serve as an effective delivery route for the treatment of related diseases.

#### 2.1.3 Naringenin

Naringenin is a flavonoid naturally found in citrus fruits such as grapefruit, orange, and lemon. It exhibits antioxidant, anti-inflammatory, neuroprotective, and anti-cancer properties. Oxidative stress plays a critical role in the pathogenesis of Parkinson’s disease. Naringenin, a potent antioxidant, has emerged as a promising therapeutic candidate for the treatment of Parkinson’s disease (Md et al., 2019) suggests that naringenin restores levels of neurotransmitters, including serotonin, epinephrine, and dopamine, by inhibiting monoamine oxidase A. An imbalance in these neurotransmitters is considered a major factor in depression (Qizilbash et al., 2022) a mouse model of allergic rhinitis, naringenin has been shown to significantly reduce the levels of serum IgE, IL-4, and IL-5, thereby ameliorating the symptoms of allergic rhinitis (Şahin et al., 2021) anti-tumor research, it has been shown to effectively inhibit lung cancer cell proliferation through increased reactive oxygen species (ROS) production and activation of the adenosine monophosphate-activated protein kinase (AMPK) signaling pathway. This process triggers autophagy, modulates the cell cycle, and promotes apoptosis. In a mouse model of lung cancer, this approach demonstrated significant anti-tumor effects (Chang et al., 2024). Naringenin has low bioavailability via conventional routes of administration, mainly due to the first-pass effect and limited solubility. Identifying novel delivery routes to improve its targeting and bioavailability remains a major focus of current research.

#### 2.1.4 Quercetin

Quercetin is a flavonoid naturally found in a variety of fruits, vegetables, leaves, and grains. It possesses multiple biological activities, including antioxidant, anti-inflammatory, and neuroprotective effects. Quercetin exerts neuroprotective effects through the upregulation of p-Akt expression, downregulation of p-ASK1, p-JNK3, and cleaved caspase-3 expression, and inhibition of neuronal apoptosis (Pei et al., 2016). Modulation of the mTOR pathway has been shown to reduce inflammation, apoptosis, and oxidative stress following spinal cord injury, while promoting axonal regeneration (Wang et al., 2022). In another study, quercetin-loaded nanoparticles were prepared and combined with a spinal cord decellularized scaffold to treat spinal cord injury and restore function after injury. They played a role in anti-inflammation, anti-oxidation, and neuroprotection (Ebrahimi et al., 2024), demonstrating significant potential in the treatment of spinal cord injury. In studies using an AR mouse model, quercetin significantly alleviated allergic symptoms and reduced serum IgE levels (Sagit et al., 2017). It regulates immune responses, stabilizes Th1/Th2 cell balance, reduces IgE antibody release, and alleviates allergic symptoms by modulating inflammatory pathways (Jafarinia et al., 2020).

#### 2.1.5 Hesperidin

Hesperidin is a natural bioflavonoid classified as a flavonoid glycoside with moderate water solubility. It is primarily found in the peel and pulp of citrus fruits, including oranges, lemons, and grapefruits. Hesperidin possesses antioxidant, anti-inflammatory, cardioprotective, anti-allergic, and neuroprotective properties. Hesperidin suppresses the receptor for advanced glycation end products (RAGE) activity and NF-κB signaling, while activating the Akt/Nrf2 pathway, thereby reducing inflammation and oxidative stress (Hong and An, 2018). The antioxidant mechanism of hesperidin involves the upregulation of Nrf2 and heme oxygenase-1 (HO-1), suppression of apoptosis, ROS and malondialdehyde (MDA) production, and enhancement of superoxide dismutase (SOD) and glutathione (GSH) expression (Muhammad et al., 2019). Hesperidin is one of the research drugs currently under investigation for the treatment of neurodegenerative diseases. In an experimental study in rats with allergic rhinitis, hesperidin significantly reduced the levels of IgE, IL-5, and IL-13 and modulated the oxidative balance. In addition, hesperidin showed comparable efficacy to desloratadine in suppressing TNF-α expression (Kilic et al., 2019).

#### 2.1.6 Kaempferol

Kaempferol is a flavonoid found naturally in various plants, including broccoli, apples, aloe, saffron, as well as in certain foods and Chinese herbs. It possesses antioxidant, anti-inflammatory, anti-tumor, and cardioprotective properties. Evidence from the literature suggests that kaempferol exerts neuroprotective effects by regulating the expression of apoptosis-related proteins. It also has anti-inflammatory effects by suppressing the production and mRNA expression of pro-inflammatory factors (Dong et al., 2023). Kaempferol exerts anti-allergic effects through the regulation of IL-32 and TSLP production, as well as caspase-1 activity, with potential applications in the treatment of various allergic diseases.

#### 2.1.7 Luteolin

Luteolin is a flavonoid found naturally in several traditional Chinese herbs and edible plants, including perilla leaves, carrots, and peppers. It possesses multiple biological activities, such as anti-inflammatory, antioxidant, anti-allergic, and neuroprotective effects. Studies in AR mouse models, as reported in the research literature, have shown that luteolin can reduce levels of IgE and IgG while suppressing the production of pro-inflammatory cytokines, such as the Th2 cytokine IL-4, thereby modulating mast cell-mediated inflammatory responses (Liang et al., 2020). By regulating the TLR4/NF-κB pathway, it ameliorates inflammatory responses and restores the Th1/Th2 immune balance in AR mice (Dong et al., 2021). Luteolin inhibits the expression of anti-apoptotic proteins and enhances pro-apoptotic signaling through multiple pathways. It also activates endoplasmic reticulum (ER) stress-related proteins, increases ROS levels in glioblastoma cells, and impairs their resistance to oxidative stress, thereby inducing apoptosis (Imran et al., 2019; Farooqi et al., 2020). In summary, luteolin has significant potential in anti-cancer, anti-allergic, and anti-inflammatory therapies.

#### 2.1.8 Mangiferin

Mangiferin is a natural flavonoid compound found mainly in mangoes. It possesses multiple biological activities, including antioxidant, anti-inflammatory, and anti-cancer effects; antiviral and antibacterial properties; as well as neuroprotective and anti-allergic effects. In studies evaluating the therapeutic potential of mangiferin in allergic rhinitis, results from an OVA-induced mouse model showed that mangiferin significantly alleviated allergic symptoms, as evidenced by reduced nasal rubbing, sneezing, and eosinophil and mast cell infiltration. In addition, mangiferin exhibits anti-inflammatory, antioxidant, and anti-allergic properties by regulating the Th1/Th2 immune response, inhibiting the NF-κB pathway, and activating the Nrf2/HO-1 antioxidant pathway (Liu et al., 2021). Evidence from other studies indicates that mangiferin can cross the BBB and protect neurons against oxidative damage through free radical scavenging and oxidative stress reduction. It demonstrates superior antioxidant activity compared to traditional antioxidants like vitamins C and E. Furthermore, mangiferin prevents neuronal death by preserving neuronal ATP levels, suppressing cytochrome c release, and inhibiting caspase activation (Walia et al., 2021). The neuroprotective and antioxidant properties of mangiferin suggest its potential as a novel therapeutic candidate for neurodegenerative diseases.

#### 2.1.9 Apigenin

Apigenin is a natural flavonoid found in several plants including celery, chamomile and thyme. It exhibits diverse biological activities, with significant potential demonstrated particularly in anti-allergic, antioxidant, anti-inflammatory and neuroprotective applications (Kashyap et al., 2022; Rahimi et al., 2022). In a mouse model of allergic rhinitis, it exerts anti-allergic effects by suppressing the Th2 response, thereby reducing IgE and histamine levels, inhibiting the TLR4/MyD88/NF-κB pathway to attenuate inflammation, and promoting Th1 activation while restoring the Th1/Th2 immune balance (Li et al., 2023a; Chen et al., 2020a).

### 2.2 Alkaloids and polyphenols

Following flavonoids, alkaloids and polyphenols represent another class of natural products with significant potential for treating neuroinflammation and allergies via the nasal route. Alkaloids are a class of nitrogen-containing organic compounds known for their diverse biological activities and significant medicinal value. These compounds are clinically important in applications such as analgesic, antimalarial, antitumor, stimulant, sedative, and antibacterial treatments.

Polyphenols are potent antioxidants found naturally in a wide range of fruits and vegetables. In addition to their antioxidant properties, polyphenols exhibit anti-atherosclerotic, hypolipidemic, and antibacterial activities (Table 2). Through diverse mechanisms like modulating NF-κB, alkaloids and polyphenols are highly promising candidates for nasal delivery to treat allergic and neurodegenerative disorders.

**TABLE 2:** Compounds Structure, Bioactivities, and Molecular mechanism of Alkaloids and Polyphenols.

Types	Name	Compounds structure	Bioactivities	Molecular mechanism	Reference
Alkaloids	Berberine	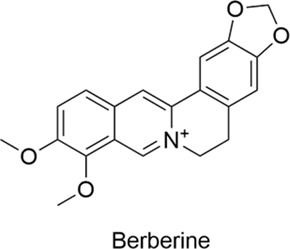	Antiallergic Antioxidant Anti-inflammatory Neuroprotective Cardioprotective	Th2↓ → IgE↓, GATA-3↓, IL-4↓, IL-13 mRNA↓, Eosinophils↓ AMPK↑, SIRT1↑, LDLR↑, PCSK9↓, PTP1B↓	Kim et al. (2015), Feng et al. (2019), Kong et al. (2020)
	Piperine	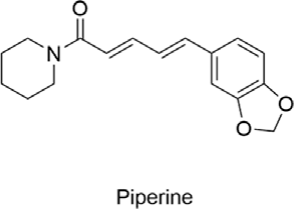	Antiallergic Antioxidant Anti-inflammatory Neuroprotective Cardioprotective	P2RX4↑ → Autophagic Flux↑ → Autophagosome-Lysosome Fusion↑ →Pathological SNCA (α-Synuclein) Degradation↑ Histamine↓, IgE↓, IL-6↓, IL-1β↓, NO↓	Li et al. (2022), Aswar et al. (2015)
Polyphenols	Resveratrol	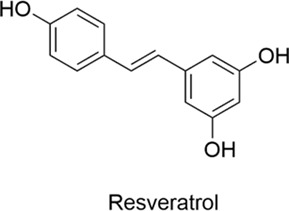	Antiallergic Antioxidant Anti-inflammatory Neuroprotective Cardioprotective	IL-4↓, IL-5↓, PGDS↓, LTC4S↓ 5-LOX↓ NF-κB↓ → Apoptosis↓, Oxidative Stress↓ IgE↓, IL-4↓, TNF-α↓, Eosinophils↓	Kim et al. (2013), Andrade et al. (2018), Seo et al. (2018), Lv et al. (2018), Zhang et al. (2020b)
	curcumin	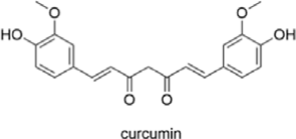	Antiallergic Antioxidant Anti-inflammatory Neuroprotective Cardioprotective	1. Eosinophils↓, IL-13↓, Mucus Overproduction↓, Oxidative Stress↓ 2. Th17↓, Cytotoxic T Cells↓, Th2 Signaling Pathway↓ 3. NF-κB↓, COX↓, LOX↓→ Pro-inflammatory Cytokines↓, Prostaglandins↓	Chen et al. (2018), Sanidad et al. (2019)

#### 2.2.1 Berberine

Berberine, an alkaloid derived from plants of the Berberidaceae family, is found in several traditional Chinese medicinal herbs, including Coptis chinensis, Phellodendron amurense, Stephania tetrandra, and Uncaria rhynchophylla. Due to its diverse biological activities, berberine has been shown to have multiple effects, such as antibacterial, anti-inflammatory, anti-tumor, glucose-lowering, and lipid-regulating properties. According to the literature, in experimental mice with allergic rhinitis treated with berberine, serum IgE, GATA-3, IL-4, and IL-13 mRNA levels, as well as tissue eosinophil numbers, were significantly reduced. These results suggest that berberine may inhibit the Th2 response. In addition, berberine treatment was found to significantly increase IL-10 levels (Kim et al., 2015). These findings highlight its significant potential as an immunomodulator for the treatment of allergic inflammation. Recent studies have consistently shown that berberine has multiple therapeutic targets, including AMPK, SIRT1, LDLR, PCSK9, and PTP1B (Feng et al., 2019; Kong et al., 2020), opening up novel therapeutic approaches for cardiovascular and metabolic diseases such as atherosclerosis and diabetes.

#### 2.2.2 Piperine

Piperine alkaloids are naturally occurring alkaloids derived from the pepper plant that exhibit diverse pharmacological activities, such as antioxidant, anti-inflammatory, anticancer, and antibacterial effects (Haq et al., 2021). Research has shown that piperine enhances autophagic flux through activation of the P2RX4 receptor, facilitates autophagosome-lysosome fusion, and thereby accelerates autophagic degradation of pathological SNCA (alpha-synuclein), exerting a neuroprotective effect. These findings highlight its potential as a therapeutic agent for Parkinson’s disease (Li et al., 2022). Another study demonstrated the significant efficacy of piperine in alleviating the symptoms of allergic rhinitis. In a mouse model, piperine effectively alleviated allergic symptoms, including sneezing and nasal rubbing, and reduced inflammatory markers, including histamine, IgE, IL-6, IL-1β, and NO levels. These results highlight its potent anti-allergic and anti-inflammatory properties. Pathological analysis of nasal mucosal tissue in mice showed that piperine-treated mice had reduced eosinophil migration and infiltration compared to the allergic rhinitis control group, further highlighting the therapeutic potential of piperine in allergic rhinitis (Aswar et al., 2015).

#### 2.2.3 Resveratrol

Resveratrol is a natural polyphenol found in many plants, including grape skins, red wine, blueberries and peanuts. It has cardiovascular protective, antioxidant, neuroprotective and anti-inflammatory properties. Studies have shown that both low and high doses of resveratrol significantly reduce the expression of IL-4, IL-5, prostaglandin D synthase and leukotriene C4 synthase. In addition, high doses of resveratrol strongly inhibit the production of 5-lipoxygenase (Kim et al., 2013) its potential as a therapeutic agent for inflammation and allergic reactions. Evidence from the literature suggests its potential to treat conditions such as Alzheimer’s disease through NF-κB blockade, apoptosis inhibition and antioxidant activity (Andrade et al., 2018; Seo et al., 2018) in allergic rhinitis have shown that resveratrol can effectively relieve allergy symptoms and significantly reduce inflammatory markers associated with allergic rhinitis, including blood levels of IgE, IL-4, TNF-α and eosinophils (Lv et al., 2018; Zhang et al., 2020b).

#### 2.2.4 Curcumin

Curcumin is a natural polyphenolic compound primarily derived from the rhizomes of Curcuma longa, a member of the ginger family. It possesses pharmacological properties, including antioxidant, anti-inflammatory, antibacterial, and anti-neurodegenerative activities. Evidence suggests that curcumin has potential as a therapeutic agent for allergic asthma by suppressing eosinophils, IL-13, mucus overproduction, oxidative stress, Th17 and cytotoxic T cell subsets, and Th2 pathway activity (Chen et al., 2018). Another study suggests that curcumin inhibits the NF-κB, COX, and lipoxygenase pathways, thereby reducing levels of pro-inflammatory factors and prostaglandin production (Sanidad et al., 2019). As a naturally occurring compound, curcumin is associated with fewer long-term side effects and is considered a promising candidate for the treatment of various chronic inflammation-related diseases.

### 2.3 Glycoside and phenylpropanoids

Glycosides and phenylpropanoids are also being actively explored for nasal-to-brain delivery due to their significant neuroprotective activities. Glycosides are ubiquitous in the plant kingdom. They exhibit diverse pharmacological activities, including anti-inflammatory, antioxidant, antitumor, and neuroprotective effects, making them highly valuable in medicine. The efficacy of many traditional Chinese medicines can be attributed to their glycoside content, such as ginsenosides in ginseng and glycyrrhizic acid in licorice. Modern scientific extraction and purification techniques allow these compounds to be formulated into various medicines for the treatment of a wide range of diseases.

Phenylpropanoids are a class of natural organic compounds that are widely distributed as secondary metabolites in plants, including Chinese medicinal herbs. They exhibit diverse pharmacological activities, including antioxidant, anti-inflammatory, antibacterial, and anticancer effects. Representative phenylpropanoids include gastrodin and asarinin (Table 3). With targeted neuroprotective mechanisms valuable for Alzheimer’s disease, these compounds are being paired with advanced nasal formulations to maximize brain delivery.

**TABLE 3:** Compounds Structure, Bioactivities, and Molecular mechanism of Glycoside and Phenylpropanoids.

Types	Name	Compounds structure	Bioactivities	Molecular mechanism	Refs
Glycosides	Geniposide	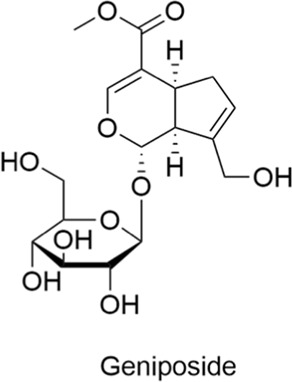	Antiallergic Antioxidant Anti-inflammatory Neuroprotective Cardioprotective	1. Aβ↓ 2. Tau hyperphosphorylation↓, Synaptic loss↓, Memory impairment↓ 3. GLP-1R↑ → Synapse growth↑ 4. P2Y14↓ → Inflammation↓, Pro-inflammatory factors↓ 5. IL-4↓, IL-5↓, IL-17↓ → IL-2↑, INF-γ↑ → CD4^+^ Tregs↓, Foxp3+ Tregs↓ → Immune tolerance↑	Ran et al. (2021), Liu et al. (2022), Li et al. (2016a), Zhang et al. (2019a)
Phenylpropanoids	Gastrodin	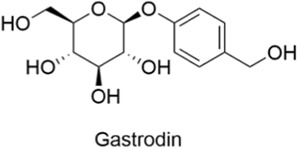	Antiallergic Antioxidant Anti-inflammatory Neuroprotective Cardioprotective	1. TNF-α↓, IL-1β↓ → Free radicals↓, Apoptosis↓ 2. TLR4↓, TRAF6↓, NF-κB↓ → Neuroinflammation↓, Microglial overactivation↓	Yao et al. (2020), Yang et al. (2022), Wang et al. (2024a)
	Asarone	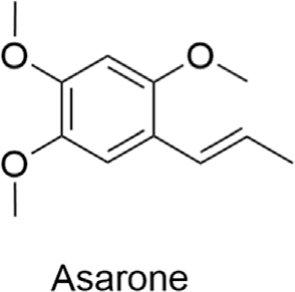	Antiallergic Antioxidant Anti-inflammatory Neuroprotective Cardioprotective	1. Neuroprotective pathways↑ → Antioxidant effects↑, Anti-inflammatory effects↑ 2. Autophagy↑ → Protein aggregation↓, ER stress↓	Xiao et al. (2019), Wang et al. (2019), Balakrishnan et al. (2022)

#### 2.3.1 Geniposide

Geniposide is a cyclic enol ether glycoside primarily derived from gardenia and found in various traditional Chinese medicinal herbs. This compound has attracted considerable attention for its diverse pharmacological activities, such as anti-inflammatory, antioxidant, anti-diabetic, and neuroprotective properties. Studies suggest that geniposide inhibits the production of amyloid beta protein (Aβ), thereby slowing the formation of amyloid plaques. It also appears to inhibit the over-phosphorylation of tau protein, reducing synaptic loss and preventing memory impairment. Geniposide alleviates mitochondrial oxidative stress and chronic inflammation, while promoting neuronal synapse growth through activation of the GLP-1 receptor pathway (Ran et al., 2021; Liu et al., 2022). Therefore, geniposide exhibits promising therapeutic effects for Alzheimer’s disease treatment. It is also capable of inhibiting downstream inflammatory signaling pathways and suppressing the release of pro-inflammatory factors, thereby mitigating inflammation (Li et al., 2016a; Liu et al., 2022). In a mouse model of allergic rhinitis, treatment with geniposide significantly reduced serum levels of IL-4, IL-5, and IL-17. It promoted immune tolerance and alleviated allergic symptoms by upregulating IL-2 and INF-γ levels while reducing CD4^+^ and Foxp3+ Treg cell counts (Zhang et al., 2019a).

#### 2.3.2 Gastrodin

Gastrodin, a phenylpropanoid compound, is primarily extracted from the traditional Chinese medicinal herb Gastrodia elata and serves as its main active ingredient. This compound exhibits multiple pharmacological activities, such as antioxidant, anti-inflammatory, neuroprotective, antidepressant, and antiepileptic effects. Evidence from the research literature suggests that gastrodin may protect cells from damage induced by ROS and reactive nitrogen species, thereby exerting neuroprotective and anti-inflammatory effects (Peng et al., 2015; Yang et al., 2022). It also modulates the expression of pro-inflammatory cytokines, including TNF-α and IL-1β, scavenges free radicals, and inhibits apoptosis (Yao et al., 2020; Yang et al., 2022). In Alzheimer’s disease research, studies have shown that gastrodin reduces neuroinflammatory responses and microglial overactivation in the central nervous system of AD models by regulating the TLR4/TRAF6/NF-κB pathway, thereby contributing to its therapeutic effects against AD (Wang et al., 2024a).

#### 2.3.3 Asarone

Asarone is a natural phenolic compound primarily derived from plants such as acorus calamus and asarum forbesii. Depending on the plant from which it is extracted, asarone may be known by different names, such as asarone and calamon. This compound exists in two isomeric forms: α-asarone and β-asarone. It has a wide range of biological effects, including antioxidant, anti-inflammatory, anti-apoptotic, anti-cancer, and neuroprotective properties. In the treatment of neurological disorders, asarone has been shown to protect nerve cells by activating neuroprotective pathways and exerting antioxidant and anti-inflammatory effects (Xiao et al., 2019; Wang et al., 2019; Balakrishnan et al., 2022). In addition, asarone may reduce protein aggregation and ER stress by regulating autophagy (Wang et al., 2019; Balakrishnan et al., 2022), which plays a critical role in regulating the progression of neurodegenerative diseases.

### 2.4 Terpenes and polysaccharides

Unlike purely therapeutic agents, terpenes and polysaccharides can also function as critical enhancers for nasal drug delivery systems. Terpenes are natural organic compounds that are widely distributed in nature, primarily derived from plants, especially those that produce essential oils. These compounds exhibit various biological activities, including anti-inflammatory, antibacterial, antioxidant, analgesic, and anticancer effects, and are widely used in medicine, cosmetics, food additives, and aromatherapy.

A natural polysaccharide such as Chitosan is derived from the deacetylation of chitin, a substance commonly found in the shells of crustaceans, such as shrimp and crabs. It exhibits various biological activities, including mucoadhesive, antioxidant, and antitumor effects. It is widely used in mucosal drug delivery and nanomedicine (Table 4). Terpenes and polysaccharides exemplify the diverse roles of natural products as permeation enhancers and foundational biomaterials for optimizing nasal delivery.

**TABLE 4:** Compounds Structure, Bioactivities, and Molecular mechanism of Terpenes and Polysaccharides.

Types	Name	Compounds structure	Bioactivities	Molecular mechanism	Refs
Terpenes	Borneol	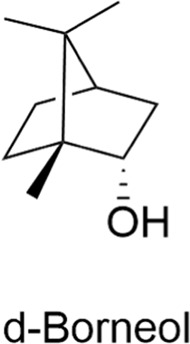	Increasing pinocytosis Anti-inflammatory Antioxidant Neuroprotective	1. Efflux transporters↓→Drug permeability↑ 2. Pinocytosis↑ in brain capillary endothelial cells → Active drug uptake↑	Kulkarni et al. (2021), Zhang et al. (2017b), Chen et al. (2010)
	Crocetin	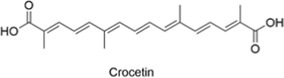	Antiallergic Antioxidant Anti-inflammatory Neuroprotective Cardioprotective	1. STK11/LKB1↑→AMPK pathway↑→Autophagy↑ in N9 microglia and primary neurons → Aβ clearance↑, Memory function↑ 2. NOX2↓ → Early ROS production↓ RNF146-mediated HK-I ubiquitination↓ (in late Parthanatos) → Mitochondrial protection↑ 3. Local cerebral blood flow↑ → Neuronal differentiation↑	Wani et al. (2021), Wu et al. (2023), Liu et al. (2023)
Polysaccharides	Chitosan	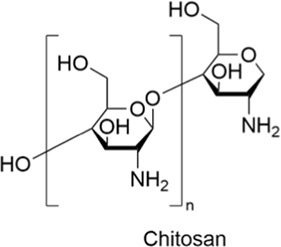	Antiallergic Antioxidant Anti-inflammatory Neuroprotective Cardioprotective	1. Negative charge on mucosal surface → Electrostatic interaction↑ → Drug bioavailability↑	Jafernik et al. (2023)

#### 2.4.1 Natural borneol

Borneol is a terpene compound primarily derived from the dipterocarpus family, including Dipterocarpus species and pine trees. Its primary function is to increase the permeability of drugs across the BBB. Studies have shown that borneol can inhibit ATP-binding cassette transporters, also known as efflux transporters, which normally prevent drugs from entering the brain from the bloodstream. By inhibiting these transporters, borneol may increase drug permeability (Kulkarni et al., 2021; Zhang et al., 2017b). Furthermore, borneol can increase pinocytosis in brain capillary endothelial cells, thereby enhancing the cells' ability to actively take up drugs (Chen et al., 2010). In conclusion, borneol has significant potential to facilitate the passage of drugs across the blood-brain barrier, enhance drug efficacy, and improve drug delivery.

#### 2.4.2 Crocetin

Crocetin is a carotenoid derivative extracted and purified from the traditional Chinese herb saffron (Crocus sativus). It has antioxidant, anti-inflammatory, neuroprotective, apoptosis-inducing, and lipid-lowering properties (Hashemi and Hosseinzadeh, 2019; Cerdá-Bernad et al., 2022). Recent studies have shown that crocetin activates the STK11/LKB1-mediated AMPK pathway, induces autophagy in N9 microglial cells and primary neurons, significantly increases Aβ clearance efficiency in N9 cells, and improves cognitive function (Wani et al., 2021). Parthanatos is a form of cell death resulting from mitochondrial dysfunction in ischemic stroke (Fricker et al., 2018; Tuo et al., 2022). Crocetin reduces early ROS production by inhibiting NOX2 activity and protects mitochondria by inhibiting E3 ligase RNF146-mediated ubiquitination of HK-I during the late stage of parthanatos (Wu et al., 2023). Crocetin also increases local cerebral blood flow and promotes neuronal differentiation. It helps to reverse neuronal apoptosis caused by ischemic stroke and promotes the repair of damaged tissue (Liu et al., 2023), showing great potential in research into ischemic stroke, Alzheimer’s disease, and other disorders.

#### 2.4.3 Chitosan

Chitosan is a natural polysaccharide derived from the deacetylation of chitin, commonly found in the shells of crustaceans such as shrimp and crab. It is a valuable, abundant, and renewable resource. Chitosan is widely used in mucosal drug delivery due to its excellent biocompatibility, biodegradability, low toxicity, and relatively low cost. It also exhibits excellent antioxidant, antibacterial, antitumor, antimicrobial, and other biological activities (Abd El-Hack et al., 2020; Rashki et al., 2021; Perinelli et al., 2018). Research has shown that chitosan has the following remarkable properties: high molecular weight chitosan significantly enhances the absorption of compounds across the mucosal barrier; its unique cationic properties bind tightly to negatively charged drug molecules, forming stable complexes, and interact electrostatically with the negative charges on the mucosal surface (Sinha et al., 2004; Jafernik et al., 2023). This effectively prolongs the residence time of the drug in the mucosa, further increasing the bioavailability of the drug (Islam et al., 2012). It is widely used in nanomedicine delivery research and has broad potential applications in diseases such as cancer and inflammation (Mushtaq et al., 2021; Rajitha et al., 2016).

## 3 Nasal application of natural products

Nasal administration of natural products has long been studied for various diseases. With their excellent biological activities and the advantages of nasal delivery, it provides new therapeutic strategies and treatment options for many conditions.

### 3.1 Ischemic stroke

Ischemic stroke (IS) accounts for 87% of all strokes. Its pathogenesis involves neuronal death, inflammation, and neurovascular damage, resulting in severe neurological symptoms (Zhou et al., 2018). Recent studies have shown the great potential of several natural products in the treatment of IS. These natural products are associated with fewer side effects. Examples include baicalin (Liu et al., 2015b; Xiang et al., 2020), resveratrol (Yu et al., 2017; Yu et al., 2021), geniposide (Zhang et al., 2017a), and crocetin. These substances have antioxidant, anti-inflammatory, and anti-apoptotic properties (Zhu et al., 2021a; Liu et al., 2016). They promote angiogenesis, nerve regeneration, and neuroprotection (Zhu et al., 2021b; Li et al., 2023b), among a wide range of other biological activities. They play a crucial role in the prevention and treatment of IS.

Issue plasminogen activator is the only drug approved by the FDA for the treatment of IS. However, its narrow therapeutic window with in 3–4.5 h of stroke on set limits its use. Exceeding this time frame can increase the risk of hemorrhagic transformation (Niego et al., 2012). There is an urgent need to explore new treatment strategies.

**FIGURE 2:**
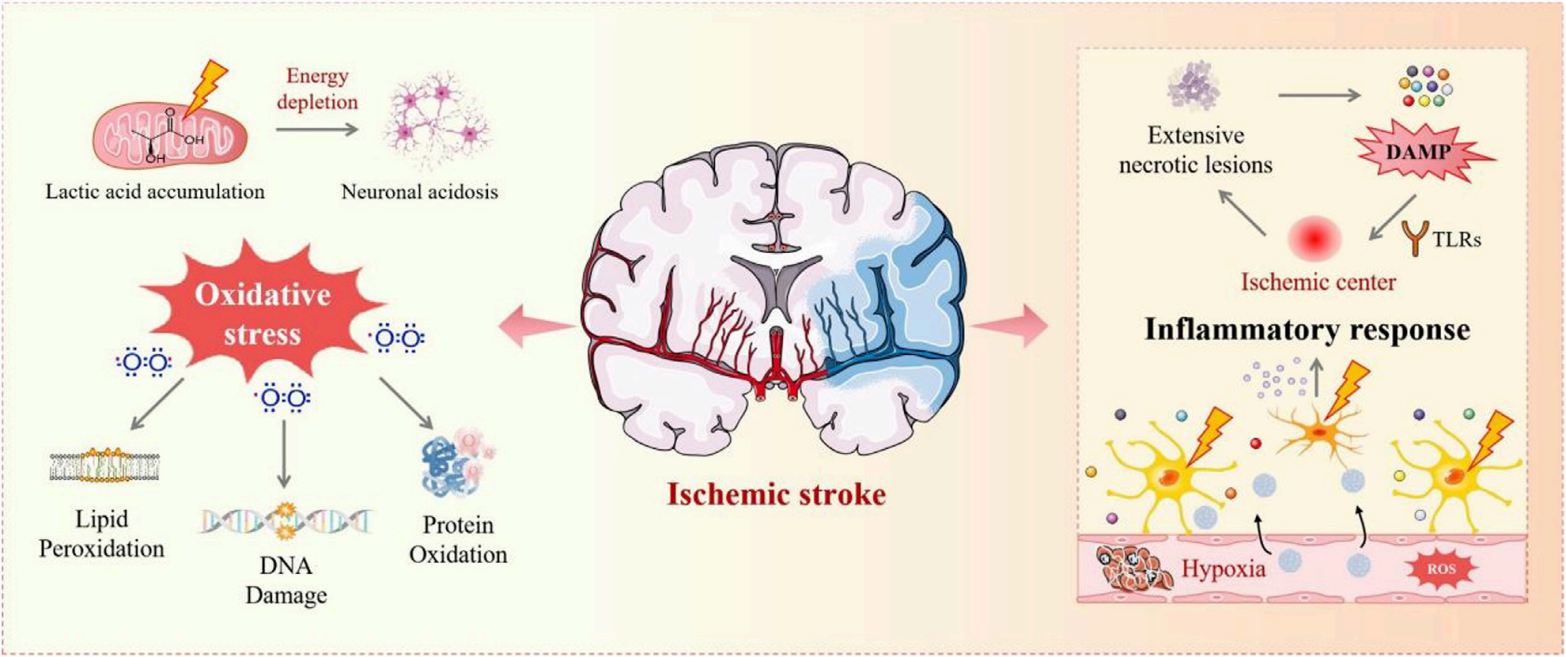
Mechanisms of ischemic stroke. This figure shows the IS process, where energy depletion and lactic acid accumulation lead to neuronal acidosis and oxidative stress. Oxidative damage causes lipid peroxidation, DNA damage, and protein oxidation, activating TLRs and triggering inflammation, worsening tissue damage, necrosis, hypoxia, and ROS production.

The pathological features of IS include oxidative stress and inflammation, exacerbated by ROS, chemokines, and cytokines (Chen et al., 2020b; Maida et al., 2020). Neural regeneration and protective mechanisms are also involved (Zhu et al., 2022) (Figure 2). Numerous studies have explored the therapeutic potential of natural products for these pathological features. However, the BBB prevents approximately 98% of potential CNS drugs from reaching the brain. Therefore, intranasal administration, which bypasses the BBB and improves drug targeting, has become a popular route for investigating the therapeutic effects of natural products. In their study (Yu et al., 2023), Shuang Yu et al. prepared liposomes with BA as the main component and borneol and cholic acid as additives. BA has excellent anti-inflammatory, antioxidant, and neuroprotective effects and effectively reduces the permeability of the BBB (Long et al., 2020; Liang et al., 2017). Borneol facilitates the passage of drugs across the BBB and can be incorporated into the phospholipid membrane of liposomes to improve formulation stability (Zhang et al., 2017b). In addition, CA promotes the release of brain-derived neurotrophic factor and activates the corresponding signaling pathway, thereby providing multiple protective effects to neurovascular units. Incorporating these compounds into BBC-LP liposomes improves drug permeability by exploiting the adhesive properties of the liposome and its ability to enhance drug solubility and stability (Wong et al., 2019). Data show that compared to BBC, BBC-LP significantly improves drug utilization and effectively alleviates nerve damage caused by IS and inhibits neuronal apoptosis by blocking related pathways. It is non-irritating to the nasal mucosa, ensuring safe use.

### 3.2 Alzheimer’s disease

**FIGURE 3:**
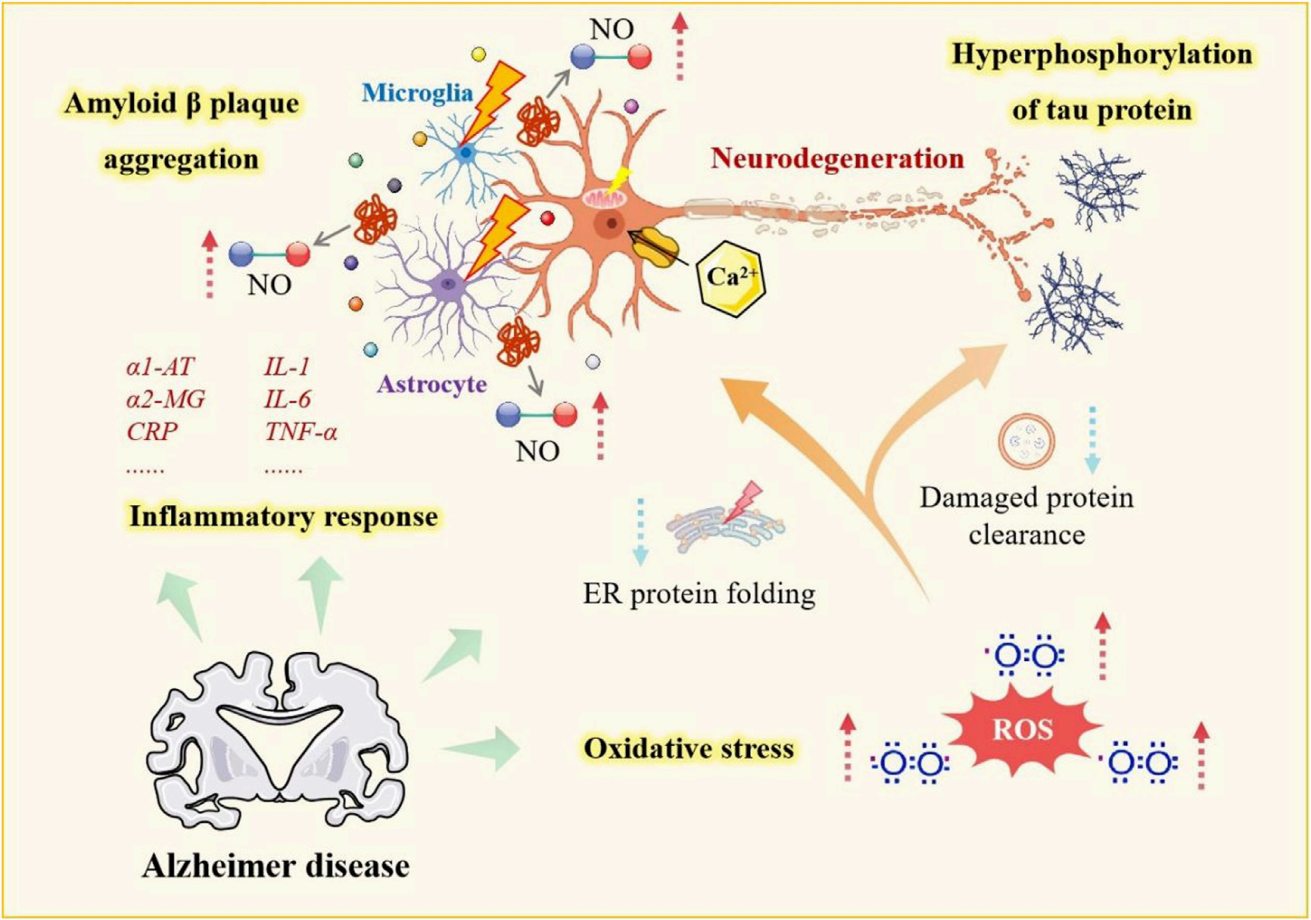
Pathogenesis of Alzheimer’s disease. This figure illustrates key mechanisms in AD, including amyloid β plaque aggregation, microglia and astrocyte-mediated inflammation, oxidative stress, tau hyperphosphorylation, and impaired protein folding. These processes disrupt neuronal function and contribute to neurodegeneration, exacerbating AD progression.

Alzheimer’s disease (AD) is an irreversible neurodegenerative disorder characterized by neuroinflammation (Mathys et al., 2019) (Figure 3). Its hallmark manifestations include progressive cognitive decline, memory loss, and central/peripheral nervous system dysfunction. According to the amyloid hypothesis, the deposition of Aβ and the formation of neurofibrillary tangles from abnormal tau protein phosphorylation are key pathological features (Mawuenyega et al., 2010). AD is closely associated with inflammation and oxidative damage, underscoring the need to reduce Aβ, protect neurons, and alleviate neuroinflammation.

Cholinesterase inhibitors (ChEIs) and NMDA receptor antagonists, such as donepezil, lisinopril, galantamine, and memantine, are the most common FDA-approved treatments but only address symptoms and can cause side effects like bradycardia and extrapyramidal symptoms, without directly targeting Aβ deposition. Various natural products, such as crocetin (Wani et al., 2021; Tong et al., 2024; Perteghella et al., 2021), gastrodin (Yao et al., 2020; Wang et al., 2024a), gardenoside (Zhang et al., 2017a; Wang et al., 2017), resveratrol (Seo et al., 2018; Andrade et al., 2018; Kotta et al., 2021) demonstrate neuroprotective, antioxidant, and anti-inflammatory properties. Crocetin inhibits β- and γ-secretase, thereby reducing Aβ production (Chalatsa et al., 2019). It also enhances Aβ clearance via cathepsin B, modulates pro-/anti-inflammatory factors (Zhang et al., 2018; Batarseh et al., 2017), and induces autophagy through STK11-AMPK (Wani et al., 2021). Intranasal silk fibroin/crocetin nanoparticles can cross the BBB, improve brain targeting, and harness their antioxidant potential (Perteghella et al., 2021), offering promise in AD therapy.

Ginsenosides have been shown to inhibit inflammation and the production and aggregation of Aβ, thereby protecting neurons from damage (Liu et al., 2018; Dai et al., 2011; El Menyiy et al., 2024; Wang et al., 2024b). Wang et al. reported that gastrodin inhibits both the TLR4/TRAF6/NF-κB pathway and Stat3 signaling, reducing microglial activation, pro-inflammatory responses, and neuronal damage in neurodegenerative diseases such as AD (Wang et al., 2024a). Cai et al. developed an *in situ* gel composed of gastrodin and deacetylated gellan gum (DGG), which rapidly transitions from solution to gel upon intranasal administration, forming a stable carrier that extends gastrodin’s residence time (Cai et al., 2011). In AD, the deposition of Aβ can damage neuronal mitochondria through multiple pathways, leading to mitochondrial dysfunction (Götz et al., 2011; Zhao et al., 2016). Additionally, geniposide alleviates Aβ-induced mitochondrial dysfunction, oxidative stress, and neuroinflammation by downregulating mTOR signaling and promoting autophagy (Zhang et al., 2021; Zhang et al., 2019b; Zhang et al., 2020c). Wang et al. formulated a nasal *in situ* gel containing poloxamers, hydroxypropylmethylcellulose, and borneol, which enhances geniposide permeation and adhesion at nasal temperature (Lu et al., 2011). This approach improves geniposide bioavailability, prolongs its duration in the nasal cavity, and minimizes mucociliary clearance and enzymatic degradation (Wang et al., 2017; Agrawal et al., 2020).

Resveratrol suppresses NF-κB signaling, reducing inflammation and immune hyperactivation (Seo et al., 2018). Nanoemulsions were prepared using coconut oil, poloxamer, and Cremophor EL. Coconut oil imparts good solubility and stability to the formulation. Poloxamer transforms into a gel at body temperature, increasing the drug retention time and absorption efficiency in the nasal cavity. Cremophor EL acts as an absorption enhancer (Li et al., 2016b), improving drug bioavailability, brain targeting, and facilitating BBB penetration (Kotta et al., 2021). This combination is expected to be an effective treatment for AD.

### 3.3 Depression

Depression is a common mental disorder that is not only one of the leading causes of disability worldwide but also contributes significantly to the global burden of disease. Risk factors include a family history of depression, childhood abuse and neglect, female gender, and daily life stress. Additionally, other medical conditions, especially metabolic and autoimmune diseases, can significantly increase the incidence of depression (Beurel et al., 2020).

The most commonly used classes of antidepressants include tricyclic antidepressants, selective serotonin reuptake inhibitors, serotonin and norepinephrine reuptake inhibitors, and norepinephrine and specific serotonin antidepressants. However, these drugs can cause side effects such as insomnia, headache, weight gain, and sexual dysfunction (Rothmore, 2020; Jha and Mathew, 2023).

**FIGURE 4:**
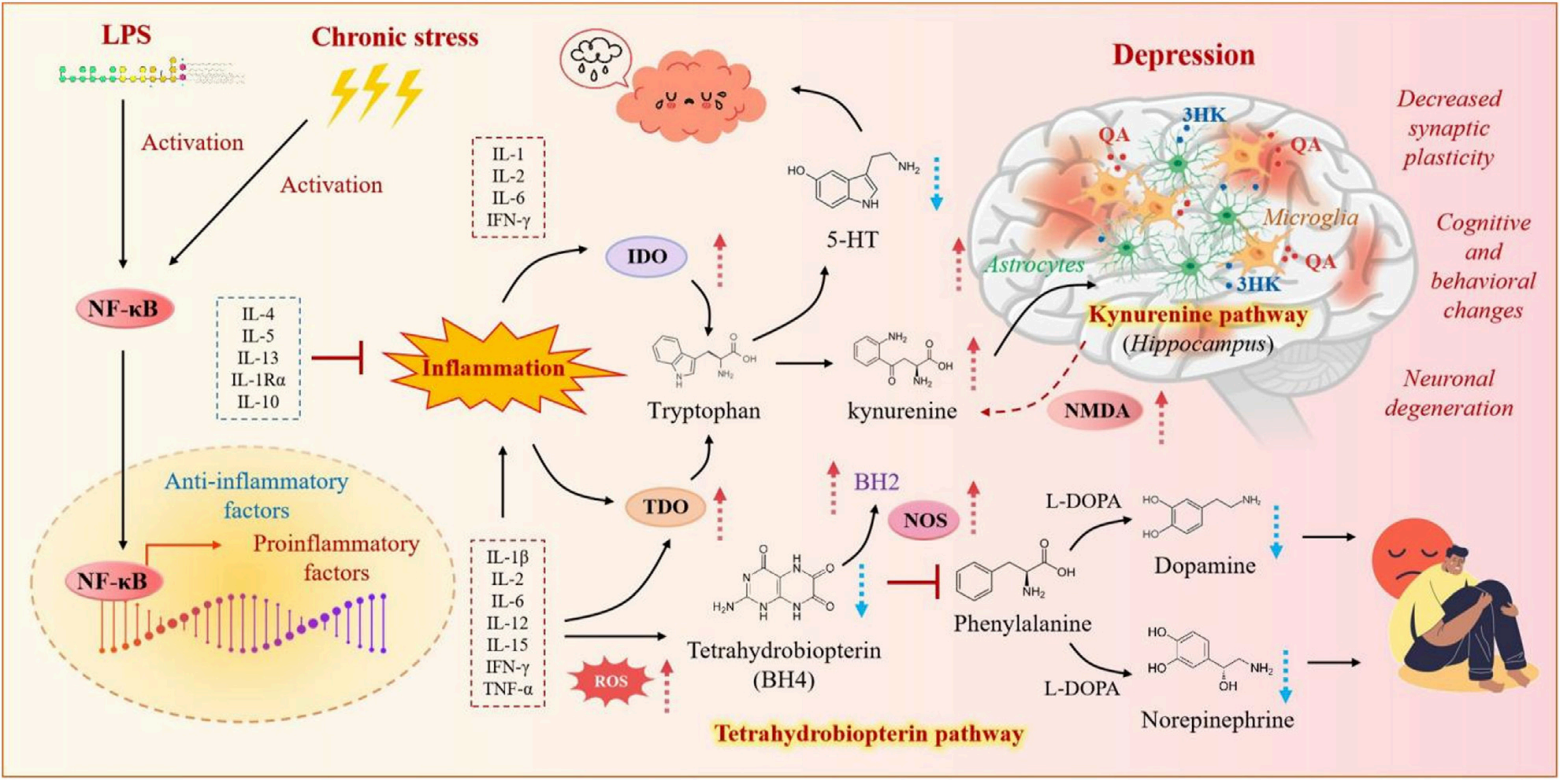
Inflammation-related neurochemical mechanisms in depression. Inflammation induces neurotoxic metabolites and reduces BH4, disrupting serotonin, dopamine, and norepinephrine synthesis, leading to neuronal dysfunction and depression.

According to studies, inflammation induces neurotoxicity and reduces BH4, impairing serotonin, dopamine, and norepinephrine synthesis, which contributes to depression (Zakaria et al., 2022) (Figure 4). Among many natural products, icariin exhibits diverse biological activities, including anti-tumor, anti-inflammatory, antioxidant, and osteoporosis benefits (Kong et al., 2015; Wei et al., 2016). Additionally, icariin may have a significant antidepressant effect by improving antioxidant status and exerting an anti-inflammatory effect on brain tissue (Liu et al., 2015a). Therefore, some studies have attempted to embed icariin-containing nanogels in poloxamer to develop an icariin nanogel self-assembled thermosensitive hydrogel system, which adheres to the nasal mucosa and prolongs drug release time, thereby improving its bioavailability. This system provides sustained release of icariin, which is delivered non-invasively to the brain via the nasal-brain pathway for rapid antidepressant action (Xu et al., 2020). Another study showed that paeoniflorin also has antidepressant potential (Wang et al., 2021). The study also used poloxamer to prepare a self-assembled thermosensitive hydrogel system of paeoniflorin nanogels, enhancing its bioavailability and brain targeting (Xu et al., 2021).

### 3.4 Allergic rhinitis

Allergic rhinitis (AR) is a common disease worldwide. It is mediated by IgE antibodies, which cause an inflammatory response in the nasal mucosa when exposed to specific allergens such as pollen, house dust mites, or animal dander. This inflammatory process is primarily characterized by nasal symptoms, including pruritus, sneezing, and congestion, and may be associated with extranasal manifestations such as conjunctivitis and coughing. The allergic reaction in rhinitis is linked to genetic factors and involves immune cells such as eosinophils, plasma cells, mast cells, and inflammatory infiltration of the mucosa (Nur Husna et al., 2022).

Current treatments for AR include allergen avoidance, immunotherapy, and drug therapy. Allergen immunotherapy is divided into subcutaneous injections and sublingual administration of allergen extract tablets. The former carries a risk of anaphylactic shock, while the latter can cause side effects such as ear itching and sore throat. Medical treatments include nasal decongestants, corticosteroids, oral antihistamines, and others. These medications also have different side effects (Moradi et al., 2024; Nur Husna et al., 2022; Small et al., 2018; Wu et al., 2019).

**FIGURE 5:**
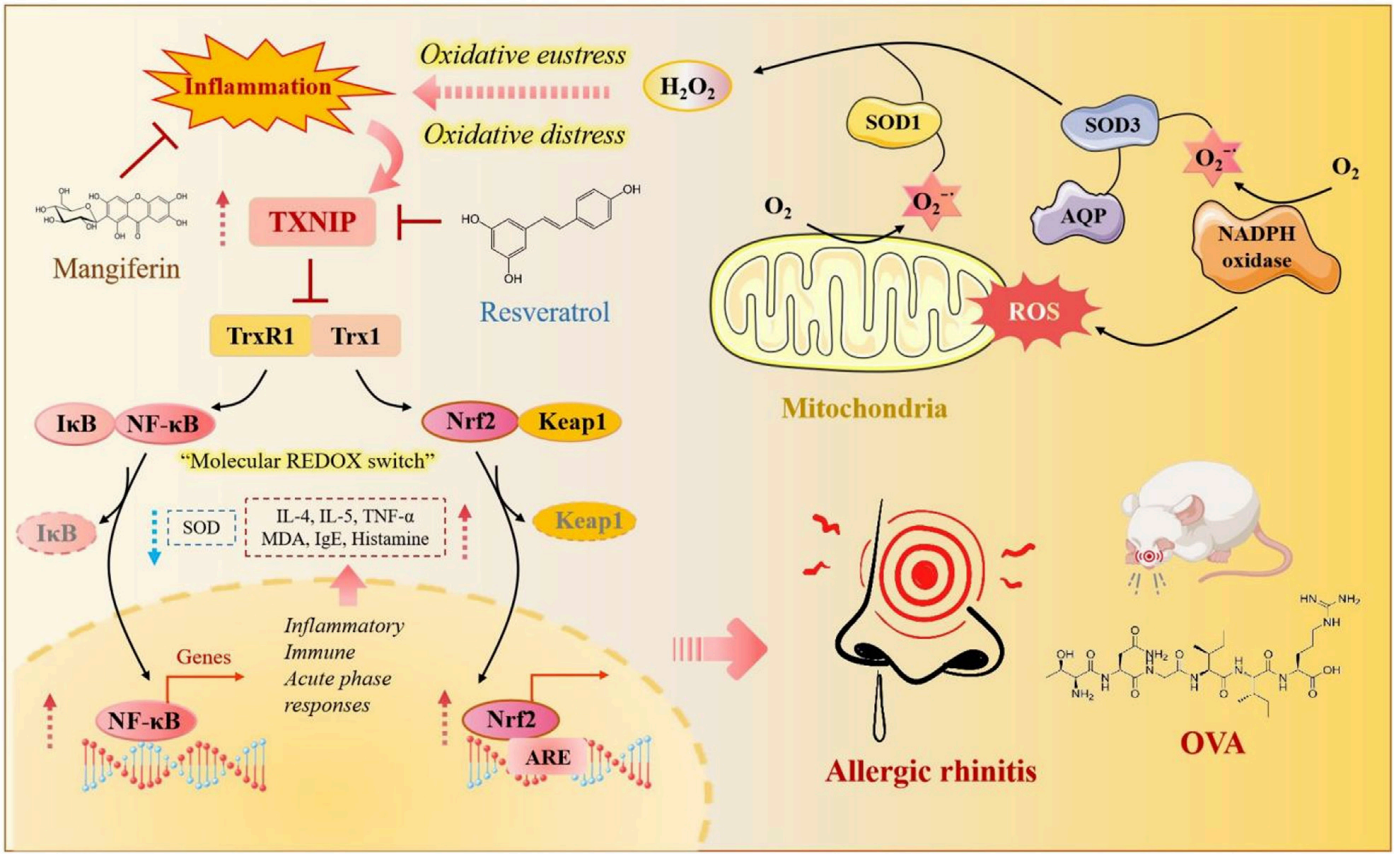
Oxidative stress–associated mechanisms in allergic rhinitis. Oxidative stress activates both the Nrf2/Keap1 and NF-κB pathways. Nrf2 signaling enhances antioxidant gene expression and cytoprotection, whereas NF-κB promotes proinflammatory gene expression, leading to inflammation and tissue damage. The interplay between these pathways contributes to the development of allergic rhinitis.

Oxidative stress activates two opposing signaling pathways: Nrf2/Keap1 and NF-κB. ROS, generated by mitochondria, NADPH oxidase, or exogenous sources, disrupt Keap1, allowing Nrf2 to enter the nucleus and induce antioxidant genes for cytoprotection. Simultaneously, oxidative stress promotes IκB degradation, activating NF-κB to upregulate proinflammatory genes. The interplay between these pathways determines the extent of inflammation or protection in oxidative stress–related diseases (Han et al., 2021) (Figure 5). However, some natural products such as berberine, baicalin, hesperidin, baicalein, quercetin, and other natural products exhibit biological activities, including anti-allergic and anti-inflammatory effects. Resveratrol significantly reduces symptoms of AR by inhibiting oxidative stress pathways. Researchers have developed a nasal spray containing resveratrol and β-glucan. Clinical trials have shown that this nasal spray effectively relieves nasal allergy symptoms in children with AR (Miraglia Del Giudice et al., 2014).

## 4 Mechanism of action

As research on natural products continues to deepen, an increasing body of evidence suggests that natural products exhibit significant biological activities in the prevention and treatment of oxidative stress, inflammatory responses, and neuronal damage. The following will provide an overview of their mechanisms of action.

### 4.1 Antioxidant

Antioxidant activity protects cells from damage caused by oxidative stress through a variety of mechanisms. Natural ingredients such as resveratrol, baicalin, and crocetin have excellent antioxidant properties, making them key resources in the fight against oxidative stress. Antioxidants directly scavenge free radicals, reducing the risk of oxidative damage. For example, resveratrol and baicalin neutralize free radicals, inhibiting oxidative processes and reducing cell damage. In addition, they enhance both enzymatic and non-enzymatic antioxidant defense mechanisms, such as increasing the activity of SOD, catalase, and GSH, thereby increasing the body’s scavenging capacity (Miguel et al., 2021; Ma et al., 2021). Moreover, natural antioxidants such as crocetin increase the body’s antioxidant capacity by activating the Nrf2/ARE pathway and promoting gene expression of antioxidant enzymes. They also protect cell membranes by inhibiting lipid peroxidation and reducing oxidative damage signaling pathways (Khoshandam et al., 2022).

In short, natural products reduce oxidative stress damage to cells and tissues and maintain good health through various mechanisms, such as neutralizing free radicals, enhancing endogenous antioxidant defenses, and activating related signaling pathways.

### 4.2 Anti-inflammatory

Natural products combat inflammation through several interconnected pathways. A primary way they work is by inhibiting the NF-κB pathway, which is a central signaling system that regulates the genes responsible for launching an inflammatory response. By suppressing this pathway, natural products like resveratrol, curcumin, and quercetin effectively reduce the body’s production of key inflammatory signals, such as TNF-α, IL-6, and IL-1β (Gupta et al., 2013). Additionally, some compounds, like curcumin, directly target specific enzymes called cyclooxygenase (COX) and lipoxygenase (LOX). These enzymes are responsible for creating potent pro-inflammatory molecules, such as prostaglandins. Inhibiting them directly lowers the levels of these triggers, thus reducing the inflammatory response. Through these combined actions, natural products can effectively manage health problems caused by both chronic and acute inflammation (Kunnumakkara et al., 2017).

Natural products also exhibit antioxidant effects that reduce oxidative stress by scavenging free radicals, thereby inhibiting inflammation caused by oxidative damage. Through these mechanisms, natural products effectively reduce inflammation and alleviate health problems caused by chronic or acute inflammation.

### 4.3 Neuroprotective and neurotrophic effects

Natural products play a key role in neuroprotection and nerve regeneration. For example, resveratrol, curcumin, and other natural products have antioxidant properties that reduce oxidative stress damage to neurons by scavenging free radicals and ROS, protecting neurons from damage in aging and disease states. On the other hand, natural products can inhibit neuroinflammatory responses and reduce inflammation-induced damage to the nervous system. For example, gastrodin reduces pro-inflammatory factors such as TNF-α and IL-6 by inhibiting the NF-κB pathway, microglial cell activation, and nerve growth factor and brain-derived neurotrophic factor secretion. This stimulates neuronal regeneration and repair, activates the PKA/CREB/BDNF pathway, and increases the expression of trophic factors, thereby reducing neuroinflammation-induced nerve damage and exerting a neuroprotective effect (Sun et al., 2023). Geniposide promotes phosphorylation of brain-derived neurotrophic factor and cAMP response element-binding protein (CREB) by enhancing cholinergic effects, thereby providing neuroprotection (Kwon et al., 2013). At the same time, some natural products promote nerve regeneration by regulating autophagy. For example, crocetin upregulates autophagy-related genes such as the AMPK/mTOR signaling pathway, thereby promoting cell autophagy, removing damaged organelles and proteins, and enhancing neuronal repair capacity (Guo et al., 2022). Quercetin exerts neuroprotective effects by increasing p-Akt expression, decreasing p-ASK1, p-JNK3, and cleaved caspase-3, and inhibiting neuronal apoptosis.

Therefore, by regulating oxidation, inflammation, apoptosis, and autophagy, these natural products collectively exert neuroprotective and neurotrophic effects, providing a promising treatment for neurodegenerative diseases and injuries.

### 4.4 The anti-allergic effects

The anti-allergic effects of natural products involve several pathways. Natural products inhibit the production of inflammatory factors such as IL-4, IL-5, and IL-13, thereby reducing the inflammatory response caused by allergies. For example, quercetin exerts its anti-inflammatory effect by inhibiting the NF-κB and MAPK pathways. Baicalin inhibits the production of inflammatory cytokines such as IL-1β, IL-6, IL-8, and TNF-α in LPS-stimulated human mast cells (Zhou et al., 2016). Anti-allergic natural products stabilize cell membranes, reduce mast cell activation, and prevent the release of allergic mediators. Baicalin is effective in this respect (Liao et al., 2021). Quercetin exerts an anti-inflammatory effect by regulating the miR-21/DMBT1/NF-κB axis and reduces LPS-mediated inflammatory damage in human nasal epithelial cells (Cheng et al., 2022).

Antioxidant effects are key mechanisms by which natural products combat allergies. Natural products such as resveratrol and quercetin reduce the intensity of allergic reactions by scavenging free radicals and reducing oxidative stress on the immune system. Many natural products inhibit the release of allergic mediators, reducing mast cell and basophil degranulation, as well as the production of chemical mediators that trigger early allergic reactions. This reduces allergic mediators such as histamine and alleviates allergic reactions. For example, SP and CGRP promote mast cell degranulation, while quercetin significantly inhibits the production of the neuropeptides SP, CGRP, and NGF in TDI-mediated allergic reactions, thereby reducing allergic symptoms (Kashiwabara et al., 2016).

## 5 Discussion

### 5.1 Advantages of natural products

Nasal administration, being non-invasive and convenient, can circumvent first-pass metabolism, enable rapid absorption, and bypass the BBB, thus dramatically improving drug bioavailability and efficacy. This is especially promising for CNS disorders. As valuable sources for new therapeutic agents, natural compounds have demonstrated anti-inflammatory, antioxidant, anti-apoptotic, and neuroprotective properties. Substances such as baicalin (Zhang et al., 2020a), geniposide (Ran et al., 2021; Liu et al., 2022), and gastrodin (Peng et al., 2015; Yang et al., 2022) significantly regulate neuroinflammation and oxidative stress. When administered via the nasal route, these natural products effectively cross the BBB, enhance targeting, and offer greater therapeutic efficacy in central nervous system disorders.

### 5.2 Challenges and future directions

Nasal drug delivery faces several challenges, including the natural cleaning mechanism of nasal cilia, which rapidly removes foreign substances, pathogens, and medications (Alsarra et al., 2010; Tai et al., 2021). This movement limits the residence time of a drug in the nasal cavity, necessitating sufficiently rapid penetration to ensure substantial or complete absorption. In addition, the nasal cavity has a limited absorptive surface, and its epithelial barrier restricts the size of penetrable substances. Compounds with a molecular weight below 1,000 Da are more easily absorbed, yet overall absorption efficiency may be significantly reduced by the viscous, negatively charged mucus layer and nasal ciliary movement (Marasini et al., 2017; Bernocchi et al., 2017).

To overcome these limitations, recent research has focused on drug delivery systems, particularly in nanomedicine. Nanocarriers, generally 10–1000 nm in size and composed of natural or synthetic polymers, include nanospheres, liposomal nanoparticles, nanoemulsions, and nanoliposomes. A fundamental challenge in nasal delivery is the rapid mucociliary clearance, which typically removes formulations in just 15–20 min. A study by Soane et al. directly addressed this by utilizing mucoadhesive chitosan particles, successfully quadrupling the nasal residence time and thus enhancing the window for drug absorption (Soane et al., 1999). Beyond normal physiological barriers, nanoparticles also tackle pathological ones. In Chronic Rhinosinusitis (CRS), for instance, Lai et al. developed mucus-penetrating nanoparticles that could rapidly penetrate the highly viscoelastic mucus layer, enabling drug delivery to previously unreachable tissue (Lai et al., 2011). Similarly, Zhang et al. targeted the notoriously resistant bacterial biofilms that complicate CRS. They engineered nanoparticles that almost completely destroyed the biofilm structure, providing a powerful new tool against chronic infections (Zhang et al., 2020).

Beyond simply reaching the target, controlling the drug’s release is crucial. Jumana et al. successfully demonstrated a desirable release profile for the corticosteroid Mometasone Furoate from PLGA nanoparticles. Their system achieved an initial burst followed by a sustained release phase, ensuring both immediate and long-term therapeutic action. The versatility of this technology also extends to diagnostics (Far et al., 2020). Broza et al. developed an innovative gold nanoparticle-based nanoarray for screening CRS from respiratory samples, which achieved high clinical utility with over 80% accuracy, specificity, and sensitivity (Broza et al., 2018).

### 5.3 Conclusion

While significant physiological barriers such as rapid mucociliary clearance and the viscous mucus layer fundamentally challenge the effectiveness of nasal drug delivery, nanoparticle-based systems have emerged as a powerful and versatile solution. As demonstrated by successful research, these technologies are not merely theoretical, they have been proven to significantly prolong drug residence time, penetrate pathological mucus barriers, eradicate resistant biofilms, and provide controlled, sustained drug release. Furthermore, their application extends beyond therapeutics into high-accuracy diagnostics. Collectively, these advancements highlight the tangible progress in the field, positioning nanotechnology as a key enabler for developing more effective therapies for various local and systemic diseases. Further research should focus on optimizing these formulations for broader clinical application and acceptance.

## References

[B1] Abd EL-Hack M. E. EL-Saadony M. T. Shafi M. E. Zabermawi N. M. Arif M. Batiha G. E. (2020). Antimicrobial and antioxidant properties of chitosan and its derivatives and their applications: a review. Int. J. Biol. Macromol. 164, 2726–2744. 10.1016/j.ijbiomac.2020.08.153 32841671

[B2] Agrawal M. Saraf S. Saraf S. Dubey S. K. Puri A. Gupta U. (2020). Stimuli-responsive *in situ* gelling system for nose-to-brain drug delivery. J. Control Release 327, 235–265. 10.1016/j.jconrel.2020.07.044 32739524

[B3] Alsarra I. A. Hamed A. Y. Alanazi F. K. EL Maghraby G. M. (2010). Vesicular systems for intranasal drug delivery. Drug Deliv. central Nerv. Syst., 175–203. 10.1007/978-1-60761-529-3_8

[B4] Andrade S. Ramalho M. J. Pereira M. D. C. Loureiro J. A. (2018). Resveratrol brain delivery for neurological disorders prevention and treatment. Front. Pharmacol. 9, 1261. 10.3389/fphar.2018.01261 30524273 PMC6262174

[B5] Anselmo A. C. Gokarn Y. Mitragotri S. (2019). Non-invasive delivery strategies for biologics. Nat. Rev. Drug Discov. 18, 19–40. 10.1038/nrd.2018.183 30498202

[B6] Aswar U. Shintre S. Chepurwar S. Aswar M. (2015). Antiallergic effect of piperine on ovalbumin-induced allergic rhinitis in mice. Pharm. Biol. 53, 1358–1366. 10.3109/13880209.2014.982299 25868617

[B7] Balakrishnan R. Cho D. Y. Kim I. S. Seol S. H. Choi D. K. (2022). Molecular mechanisms and therapeutic potential of α- and β-Asarone in the treatment of neurological disorders. Antioxidants (Basel) 11, 281. 10.3390/antiox11020281 35204164 PMC8868500

[B8] Batarseh Y. S. Bharate S. S. Kumar V. Kumar A. Vishwakarma R. A. Bharate S. B. (2017). Crocus sativus extract tightens the blood-brain barrier, reduces amyloid β load and related toxicity in 5XFAD mice. ACS Chem. Neurosci. 8, 1756–1766. 10.1021/acschemneuro.7b00101 28471166 PMC5559303

[B9] Bernocchi B. Carpentier R. Betbeder D. (2017). Nasal nanovaccines. Int. J. Pharm. 530, 128–138. 10.1016/j.ijpharm.2017.07.012 28698066

[B10] Beurel E. Toups M. Nemeroff C. B. (2020). The bidirectional relationship of depression and inflammation: double trouble. Neuron 107, 234–256. 10.1016/j.neuron.2020.06.002 32553197 PMC7381373

[B11] Broza Y. Y. Braverman I. Haick H. (2018). Breath volatolomics for diagnosing chronic rhinosinusitis. Int. J. nanomedicine 13, 4661–4670. 10.2147/IJN.S171488 30147315 PMC6097827

[B12] Bui T. T. Piao C. H. Song C. H. Lee C. H. Shin H. S. Chai O. H. (2017). Baicalein, wogonin, and Scutellaria baicalensis ethanol extract alleviate ovalbumin-induced allergic airway inflammation and mast cell-mediated anaphylactic shock by regulation of Th1/Th2 imbalance and histamine release. Anat. Cell Biol. 50, 124–134. 10.5115/acb.2017.50.2.124 28713616 PMC5509896

[B13] Cai Z. Song X. Sun F. Yang Z. Hou S. Liu Z. (2011). Formulation and evaluation of *in situ* gelling systems for intranasal administration of gastrodin. AAPS PharmSciTech 12, 1102–1109. 10.1208/s12249-011-9678-y 21879392 PMC3225523

[B14] Cerdá-Bernad D. Valero-Cases E. Pastor J. J. Frutos M. J. (2022). Saffron bioactives crocin, crocetin and safranal: effect on oxidative stress and mechanisms of action. Crit. Rev. Food Sci. Nutr. 62, 3232–3249. 10.1080/10408398.2020.1864279 33356506

[B15] Chalatsa I. Arvanitis D. A. Koulakiotis N. S. Giagini A. Skaltsounis A. L. Papadopoulou-Daifoti Z. (2019). The crocus sativus compounds trans-Crocin 4 and trans-Crocetin modulate the amyloidogenic pathway and tau misprocessing in alzheimer disease neuronal cell culture models. Front. Neurosci. 13, 249. 10.3389/fnins.2019.00249 30971876 PMC6443833

[B16] Chang T. M. Chi M. C. Chiang Y. C. Lin C. M. Fang M. L. Lee C. W. (2024). Promotion of ROS-mediated apoptosis, G2/M arrest, and autophagy by naringenin in non-small cell lung cancer. Int. J. Biol. Sci. 20, 1093–1109. 10.7150/ijbs.85443 38322119 PMC10845293

[B17] Chen X. H. Lin Z. Z. Liu A. M. Ye J. T. Luo Y. Luo Y. Y. (2010). The orally combined neuroprotective effects of sodium ferulate and borneol against transient global ischaemia in C57 BL/6J mice. J. Pharm. Pharmacol. 62, 915–923. 10.1211/jpp.62.07.0013 20636880

[B18] Chen B. L. Chen Y. Q. Ma B. H. Yu S. F. Li L. Y. Zeng Q. X. (2018). Tetrahydrocurcumin, a major metabolite of curcumin, ameliorates allergic airway inflammation by attenuating Th2 response and suppressing the IL-4Rα-Jak1-STAT6 and Jagged1/Jagged2 -Notch1/Notch2 pathways in asthmatic mice. Clin. Exp. Allergy 48, 1494–1508. 10.1111/cea.13258 30137697

[B19] Chen S. Chen G. Shu S. Xu Y. Ma X. (2019). Metabolomics analysis of baicalin on ovalbumin-sensitized allergic rhinitis rats. R. Soc. Open Sci. 6, 181081. 10.1098/rsos.181081 30891260 PMC6408364

[B20] Chen F. He D. Yan B. (2020a). Apigenin attenuates allergic responses of ovalbumin-induced allergic rhinitis through modulation of Th1/Th2 responses in experimental mice. Dose Response 18, 1559325820904799. 10.1177/1559325820904799 32165873 PMC7054738

[B21] Chen H. He Y. Chen S. Qi S. Shen J. (2020b). Therapeutic targets of oxidative/nitrosative stress and neuroinflammation in ischemic stroke: applications for natural product efficacy with omics and systemic biology. Pharmacol. Res. 158, 104877. 10.1016/j.phrs.2020.104877 32407958

[B22] Cheng J. Luo X. Q. Chen F. S. (2022). Quercetin attenuates lipopolysaccharide-mediated inflammatory injury in human nasal epithelial cells *via* regulating miR-21/DMBT1/NF-κB axis. Immunopharmacol. Immunotoxicol. 44, 7–16. 10.1080/08923973.2021.1988963 34927513

[B23] Dai J. N. Zong Y. Zhong L. M. Li Y. M. Zhang W. Bian L. G. (2011). Gastrodin inhibits expression of inducible NO synthase, cyclooxygenase-2 and proinflammatory cytokines in cultured LPS-stimulated microglia *via* MAPK pathways. PLoS One 6, e21891. 10.1371/journal.pone.0021891 21765922 PMC3134470

[B24] Dong J. Xu O. Wang J. Shan C. Ren X. (2021). Luteolin ameliorates inflammation and Th1/Th2 imbalance *via* regulating the TLR4/NF-κB pathway in allergic rhinitis rats. Immunopharmacol. Immunotoxicol. 43, 319–327. 10.1080/08923973.2021.1905659 33900898

[B25] Dong X. Zhou S. Nao J. (2023). Kaempferol as a therapeutic agent in Alzheimer's disease: evidence from preclinical studies. Ageing Res. Rev. 87, 101910. 10.1016/j.arr.2023.101910 36924572

[B26] Ebrahimi B. Mokhtari T. Ghaffari N. Adabi M. Hassanzadeh G. (2024). Acellular spinal cord scaffold containing quercetin-encapsulated nanoparticles plays an anti-inflammatory role in functional recovery from spinal cord injury in rats. Inflammopharmacology 32, 2505–2524. 10.1007/s10787-024-01478-z 38702577

[B27] EL Menyiy N. Elouafy Y. Moubachir R. Abdnim R. Benali T. Taha D. (2024). Chemistry, biological activities, and pharmacological properties of gastrodin: mechanism insights. Chem. Biodivers. 21, e202400402. 10.1002/cbdv.202400402 38573028

[B28] Far J. Abdel-Haq M. Gruber M. Abu Ammar A. (2020). Developing biodegradable nanoparticles loaded with mometasone furoate for potential nasal drug delivery. ACS omega 5, 7432–7439. 10.1021/acsomega.0c00111 32280885 PMC7144157

[B29] Farooqi A. A. Butt G. EL-Zahaby S. A. Attar R. Sabitaliyevich U. Y. Jovic J. J. (2020). Luteolin mediated targeting of protein network and microRNAs in different cancers: focus on JAK-STAT, NOTCH, mTOR and TRAIL-mediated signaling pathways. Pharmacol. Res. 160, 105188. 10.1016/j.phrs.2020.105188 32919041

[B30] Feng X. Sureda A. Jafari S. Memariani Z. Tewari D. Annunziata G. (2019). Berberine in cardiovascular and metabolic diseases: from mechanisms to therapeutics. Theranostics 9, 1923–1951. 10.7150/thno.30787 31037148 PMC6485276

[B31] Fricker M. Tolkovsky A. M. Borutaite V. Coleman M. Brown G. C. (2018). Neuronal cell death. Physiol. Rev. 98, 813–880. 10.1152/physrev.00011.2017 29488822 PMC5966715

[B32] GöTZ J. Eckert A. Matamales M. Ittner L. M. Liu X. (2011). Modes of Aβ toxicity in Alzheimer's disease. Cell Mol. Life Sci. 68, 3359–3375. 10.1007/s00018-011-0750-2 21706148 PMC3181413

[B33] Guo H. Ruan C. Zhan X. Pan H. Luo Y. Gao K. (2022). Crocetin protected human hepatocyte LO2 cell from TGF-β-Induced oxygen stress and apoptosis but promoted proliferation and autophagy *via* AMPK/m-TOR pathway. Front. Public Health 10, 909125. 10.3389/fpubh.2022.909125 35836988 PMC9273739

[B34] Gupta S. C. Patchva S. Aggarwal B. B. (2013). Therapeutic roles of curcumin: lessons learned from clinical trials. Aaps J. 15, 195–218. 10.1208/s12248-012-9432-8 23143785 PMC3535097

[B35] Han M. Lee D. Lee S. H. Kim T. H. (2021). Oxidative stress and antioxidant pathway in allergic rhinitis. Antioxidants (Basel) 10, 1266. 10.3390/antiox10081266 34439514 PMC8389336

[B36] Haq I. U. Imran M. Nadeem M. Tufail T. Gondal T. A. Mubarak M. S. (2021). Piperine: a review of its biological effects. Phytother. Res. 35, 680–700. 10.1002/ptr.6855 32929825

[B37] Hashemi M. Hosseinzadeh H. (2019). A comprehensive review on biological activities and toxicology of crocetin. Food Chem. Toxicol. 130, 44–60. 10.1016/j.fct.2019.05.017 31100302

[B38] Hong Y. An Z. (2018). Hesperidin attenuates learning and memory deficits in APP/PS1 mice through activation of Akt/Nrf2 signaling and inhibition of RAGE/NF-κB signaling. Arch. Pharm. Res. 41, 655–663. 10.1007/s12272-015-0662-z 26391026

[B39] Imran M. Rauf A. Abu-Izneid T. Nadeem M. Shariati M. A. Khan I. A. (2019). Luteolin, a flavonoid, as an anticancer agent: a review. Biomed. Pharmacother. 112, 108612. 10.1016/j.biopha.2019.108612 30798142

[B40] Islam M. A. Firdous J. Choi Y. J. Yun C. H. Cho C. S. (2012). Design and application of chitosan microspheres as oral and nasal vaccine carriers: an updated review. Int. J. Nanomedicine 7, 6077–6093. 10.2147/ijn.S38330 23271909 PMC3526152

[B41] Jafarinia M. Sadat Hosseini M. Kasiri N. Fazel N. Fathi F. Ganjalikhani Hakemi M. (2020). Quercetin with the potential effect on allergic diseases. Allergy Asthma Clin. Immunol. 16, 36. 10.1186/s13223-020-00434-0 32467711 PMC7227109

[B42] Jafernik K. Ładniak A. Blicharska E. Czarnek K. Ekiert H. Wiącek A. E. (2023). Chitosan-based nanoparticles as effective drug delivery Systems-A review. Molecules 28, 1963. 10.3390/molecules28041963 36838951 PMC9959713

[B43] Jha M. K. Mathew S. J. (2023). Pharmacotherapies for treatment-resistant depression: how antipsychotics fit in the rapidly evolving therapeutic landscape. Am. J. Psychiatry 180, 190–199. 10.1176/appi.ajp.20230025 36855876

[B44] Kashiwabara M. Asano K. Mizuyoshi T. Kobayashi H. (2016). Suppression of neuropeptide production by quercetin in allergic rhinitis model rats. BMC Complement. Altern. Med. 16, 132. 10.1186/s12906-016-1123-z 27207147 PMC4875744

[B45] Kashyap K. Shukla R. (2019). Drug delivery and targeting to the brain through nasal route: mechanisms, applications and challenges. Curr. Drug Deliv. 16, 887–901. 10.2174/1567201816666191029122740 31660815

[B46] Kashyap P. Shikha D. Thakur M. Aneja A. (2022). Functionality of apigenin as a potent antioxidant with emphasis on bioavailability, metabolism, action mechanism and *in vitro* and *in vivo* studies: a review. J. Food Biochem. 46, e13950. 10.1111/jfbc.13950 34569073

[B47] Khoshandam A. Razavi B. M. Hosseinzadeh H. (2022). Interaction of saffron and its constituents with Nrf2 signaling pathway: a review. Iran. J. Basic Med. Sci. 25, 789–798. 10.22038/ijbms.2022.61986.13719 36033950 PMC9392575

[B48] Kilic K. Sakat M. S. Yildirim S. Kandemir F. M. Gozeler M. S. Dortbudak M. B. (2019). The amendatory effect of hesperidin and thymol in allergic rhinitis: an ovalbumin-induced rat model. Eur. Arch. Otorhinolaryngol. 276, 407–415. 10.1007/s00405-018-5222-y 30488351

[B49] Kim S. W. Kim D. W. Khalmuratova R. Kim J. H. Jung M. H. Chang D. Y. (2013). Resveratrol prevents development of eosinophilic rhinosinusitis with nasal polyps in a mouse model. Allergy 68, 862–869. 10.1111/all.12132 23751068

[B50] Kim B. Y. Park H. R. Jeong H. G. Kim S. W. (2015). Berberine reduce allergic inflammation in a house dust mite allergic rhinitis mouse model. Rhinology 53, 353–358. 10.4193/Rhino15.028 26275466

[B51] Kong L. Liu J. Wang J. Luo Q. Zhang H. Liu B. (2015). Icariin inhibits TNF-α/IFN-γ induced inflammatory response *via* inhibition of the substance P and p38-MAPK signaling pathway in human keratinocytes. Int. Immunopharmacol. 29, 401–407. 10.1016/j.intimp.2015.10.023 26507164

[B52] Kong W. J. Vernieri C. Foiani M. Jiang J. D. (2020). Berberine in the treatment of metabolism-related chronic diseases: a drug cloud (dCloud) effect to target multifactorial disorders. Pharmacol. Ther. 209, 107496. 10.1016/j.pharmthera.2020.107496 32001311

[B53] Kotta S. Mubarak Aldawsari H. Badr-Eldin S. M. Alhakamy N. A. Md S. (2021). Coconut oil-based resveratrol nanoemulsion: optimization using response surface methodology, stability assessment and pharmacokinetic evaluation. Food Chem. 357, 129721. 10.1016/j.foodchem.2021.129721 33866243

[B54] Kulkarni M. Sawant N. Kolapkar A. Huprikar A. Desai N. (2021). Borneol: a promising monoterpenoid in enhancing drug delivery across various physiological barriers. AAPS PharmSciTech 22, 145. 10.1208/s12249-021-01999-8 33913042

[B55] Kunnumakkara A. B. Bordoloi D. Padmavathi G. Monisha J. Roy N. K. Prasad S. (2017). Curcumin, the golden nutraceutical: multitargeting for multiple chronic diseases. Br. J. Pharmacol. 174, 1325–1348. 10.1111/bph.13621 27638428 PMC5429333

[B56] Kwon S. H. Ma S. X. Joo H. J. Lee S. Y. Jang C. G. (2013). Inhibitory effects of Eucommia ulmoides oliv. Bark on scopolamine-induced learning and memory deficits in mice. Biomol. Ther. Seoul. 21, 462–469. 10.4062/biomolther.2013.074 24404337 PMC3879918

[B57] Laffleur F. Bauer B. (2021). Progress in nasal drug delivery systems. Int. J. Pharm. 607, 120994. 10.1016/j.ijpharm.2021.120994 34390810

[B58] Lai S. K. Suk J. S. Pace A. Wang Y. Y. Yang M. Mert O. (2011). Drug carrier nanoparticles that penetrate human chronic rhinosinusitis mucus. Biomaterials 32, 6285–6290. 10.1016/j.biomaterials.2011.05.008 21665271 PMC3130096

[B59] Li F. Li W. Li X. Li F. Zhang L. Wang B. (2016a). Geniposide attenuates inflammatory response by suppressing P2Y14 receptor and downstream ERK1/2 signaling pathway in oxygen and glucose deprivation-induced brain microvascular endothelial cells. J. Ethnopharmacol. 185, 77–86. 10.1016/j.jep.2016.03.025 26976766

[B60] Li Y. Li J. Zhang X. Ding J. Mao S. (2016b). Non-ionic surfactants as novel intranasal absorption enhancers: *in vitro* and *in vivo* characterization. Drug Deliv. 23, 2272–2279. 10.3109/10717544.2014.971196 25347689

[B61] Li R. Lu Y. Zhang Q. Liu W. Yang R. Jiao J. (2022). Piperine promotes autophagy flux by P2RX4 activation in SNCA/α-synuclein-induced parkinson disease model. Autophagy 18, 559–575. 10.1080/15548627.2021.1937897 34092198 PMC9037522

[B62] Li H. Zhang H. Zhao H. (2023a). Apigenin attenuates inflammatory response in allergic rhinitis mice by inhibiting the TLR4/MyD88/NF-κB signaling pathway. Environ. Toxicol. 38, 253–265. 10.1002/tox.23699 36350155

[B63] Li L. Yang J. H. Li C. Zhou H. F. Yu L. Wu X. L. (2023b). Danhong injection improves neurological function in rats with ischemic stroke by enhancing neurogenesis and activating BDNF/AKT/CREB signaling pathway. Biomed. Pharmacother. 163, 114887. 10.1016/j.biopha.2023.114887 37207429

[B64] Liang W. Huang X. Chen W. (2017). The effects of baicalin and baicalein on cerebral ischemia: a review. Aging Dis. 8, 850–867. 10.14336/ad.2017.0829 29344420 PMC5758355

[B65] Liang K. L. Yu S. J. Huang W. C. Yen H. R. (2020). Luteolin attenuates allergic nasal inflammation via inhibition of Interleukin-4 in an allergic rhinitis mouse model and peripheral blood from human subjects with allergic rhinitis. Front. Pharmacol. 11, 291. 10.3389/fphar.2020.00291 32256362 PMC7093717

[B66] Liao H. Ye J. Gao L. Liu Y. (2021). The main bioactive compounds of Scutellaria baicalensis georgi. For alleviation of inflammatory cytokines: a comprehensive review. Biomed. Pharmacother. 133, 110917. 10.1016/j.biopha.2020.110917 33217688

[B67] Liu B. Xu C. Wu X. Liu F. Du Y. Sun J. (2015a). Icariin exerts an antidepressant effect in an unpredictable chronic mild stress model of depression in rats and is associated with the regulation of hippocampal neuroinflammation. Neuroscience 294, 193–205. 10.1016/j.neuroscience.2015.02.053 25791226

[B68] Liu Z. Zhang L. He Q. Liu X. Okeke C. I. Tong L. (2015b). Effect of Baicalin-loaded PEGylated cationic solid lipid nanoparticles modified by OX26 antibody on regulating the levels of baicalin and amino acids during cerebral ischemia-reperfusion in rats. Int. J. Pharm. 489, 131–138. 10.1016/j.ijpharm.2015.04.049 25895718

[B69] Liu B. Li F. Shi J. Yang D. Deng Y. Gong Q. (2016). Gastrodin ameliorates subacute phase cerebral ischemia-reperfusion injury by inhibiting inflammation and apoptosis in rats. Mol. Med. Rep. 14, 4144–4152. 10.3892/mmr.2016.5785 27748849 PMC5101922

[B70] Liu Y. Gao J. Peng M. Meng H. Ma H. Cai P. (2018). A review on central nervous system effects of gastrodin. Front. Pharmacol. 9, 24. 10.3389/fphar.2018.00024 29456504 PMC5801292

[B71] Liu T. Song Y. Hu A. (2021). Neuroprotective mechanisms of mangiferin in neurodegenerative diseases. Drug Dev. Res. 82, 494–502. 10.1002/ddr.21783 33458836

[B72] Liu L. Wu Q. Chen Y. Gu G. Gao R. Peng B. (2022). Updated pharmacological effects, molecular mechanisms, and therapeutic potential of natural product geniposide. Molecules 27, 3319. 10.3390/molecules27103319 35630796 PMC9144884

[B73] Liu Z. Wang Z. Zhu Z. Hong J. Cui L. Hao Y. (2023). Crocetin regulates functions of neural stem cells to generate new neurons for cerebral ischemia recovery. Adv. Healthc. Mater 12, e2203132. 10.1002/adhm.202203132 37001492

[B74] Lobaina Mato Y. (2019). Nasal route for vaccine and drug delivery: features and current opportunities. Int. J. Pharm. 572, 118813. 10.1016/j.ijpharm.2019.118813 31678521

[B75] Long Y. Yang Q. Xiang Y. Zhang Y. Wan J. Liu S. (2020). Nose to brain drug delivery - a promising strategy for active components from herbal medicine for treating cerebral ischemia reperfusion. Pharmacol. Res. 159, 104795. 10.1016/j.phrs.2020.104795 32278035

[B76] Lu Y. Du S. Y. Chen X. L. Wu Q. Song X. Xu B. (2011). Enhancing effect of natural borneol on the absorption of geniposide in rat via intranasal administration. J. Zhejiang Univ. Sci. B 12, 143–148. 10.1631/jzus.B1000121 21265046 PMC3030959

[B77] Lv C. Zhang Y. Shen L. (2018). Preliminary clinical effect evaluation of resveratrol in adults with allergic rhinitis. Int. Arch. Allergy Immunol. 175, 231–236. 10.1159/000486959 29539616

[B78] Ma L. Wu F. Shao Q. Chen G. Xu L. Lu F. (2021). Baicalin alleviates oxidative stress and inflammation in diabetic nephropathy via Nrf2 and MAPK signaling pathway. Drug Des. Devel Ther. 15, 3207–3221. 10.2147/dddt.S319260 34321869 PMC8313380

[B79] Maida C. D. Norrito R. L. Daidone M. Tuttolomondo A. Pinto A. (2020). Neuroinflammatory mechanisms in ischemic stroke: focus on cardioembolic stroke, background, and therapeutic approaches. Int. J. Mol. Sci. 21, 6454. 10.3390/ijms21186454 32899616 PMC7555650

[B80] Marasini N. Skwarczynski M. Toth I. (2017). Intranasal delivery of nanoparticle-based vaccines. Ther. Deliv. 8, 151–167. 10.4155/tde-2016-0068 28145824

[B81] Mathys H. Davila-Velderrain J. Peng Z. Gao F. Mohammadi S. Young J. Z. (2019). Single-cell transcriptomic analysis of alzheimer's disease. Nature 570, 332–337. 10.1038/s41586-019-1195-2 31042697 PMC6865822

[B82] Mawuenyega K. G. Sigurdson W. Ovod V. Munsell L. Kasten T. Morris J. C. (2010). Decreased clearance of CNS beta-amyloid in alzheimer's disease. Science 330, 1774. 10.1126/science.1197623 21148344 PMC3073454

[B83] Md S. Alhakamy N. A. Aldawsari H. M. Asfour H. Z. (2019). Neuroprotective and antioxidant effect of naringenin-loaded nanoparticles for nose-to-brain delivery. Brain Sci. 9, 275. 10.3390/brainsci9100275 31618942 PMC6827151

[B84] Miguel C. A. Noya-Riobó M. V. Mazzone G. L. Villar M. J. Coronel M. F. (2021). Antioxidant, anti-inflammatory and neuroprotective actions of resveratrol after experimental nervous system insults. Special focus on the molecular mechanisms involved. Neurochem. Int. 150, 105188. 10.1016/j.neuint.2021.105188 34536545

[B85] Miraglia Del Giudice M. Maiello N. Capristo C. Alterio E. Capasso M. Perrone L. (2014). Resveratrol plus carboxymethyl-β-glucan reduces nasal symptoms in children with pollen-induced allergic rhinitis. Curr. Med. Res. Opin. 30, 1931–1935. 10.1185/03007995.2014.938731 24983742

[B86] Moradi S. Khazaei H. Tarlan M. Jasemi S. V. Joshi T. Aneva I. Y. (2024). Natural products for the treatment of allergic rhinitis: focus on cellular signaling pathways and pharmacological targets. Front. Pharmacol. 15, 1447097. 10.3389/fphar.2024.1447097 39403140 PMC11472003

[B87] Muhammad T. Ali T. Ikram M. Khan A. Alam S. I. Kim M. O. (2019). Melatonin rescue oxidative stress-mediated Neuroinflammation/neurodegeneration and memory impairment in scopolamine-induced amnesia mice model. J. Neuroimmune Pharmacol. 14, 278–294. 10.1007/s11481-018-9824-3 30478761

[B88] Mushtaq A. Li L. A A. GrøNDAHL L. (2021). Chitosan nanomedicine in cancer therapy: targeted delivery and cellular uptake. Macromol. Biosci. 21, e2100005. 10.1002/mabi.202100005 33738977

[B89] Niego B. Freeman R. Puschmann T. B. Turnley A. M. Medcalf R. L. (2012). t-PA-specific modulation of a human blood-brain barrier model involves plasmin-mediated activation of the Rho kinase pathway in astrocytes. Blood 119, 4752–4761. 10.1182/blood-2011-07-369512 22262761

[B90] Nur Husna S. M. Tan H. T. Md Shukri N. Mohd Ashari N. S. Wong K. K. (2022). Allergic rhinitis: a clinical and pathophysiological overview. Front. Med. (Lausanne) 9, 874114. 10.3389/fmed.2022.874114 35463011 PMC9021509

[B91] Oh H. A. Han N. R. Kim M. J. Kim H. M. Jeong H. J. (2013). Evaluation of the effect of kaempferol in a murine allergic rhinitis model. Eur. J. Pharmacol. 718, 48–56. 10.1016/j.ejphar.2013.08.045 24056122

[B92] Pei B. Yang M. Qi X. Shen X. Chen X. Zhang F. (2016). Quercetin ameliorates ischemia/reperfusion-induced cognitive deficits by inhibiting ASK1/JNK3/caspase-3 by enhancing the Akt signaling pathway. Biochem. Biophys. Res. Commun. 478, 199–205. 10.1016/j.bbrc.2016.07.068 27450812

[B93] Peng Z. Wang S. Chen G. Cai M. Liu R. Deng J. (2015). Gastrodin alleviates cerebral ischemic damage in mice by improving anti-oxidant and anti-inflammation activities and inhibiting apoptosis pathway. Neurochem. Res. 40, 661–673. 10.1007/s11064-015-1513-5 25582916

[B94] Perinelli D. R. Fagioli L. Campana R. Lam J. K. W. Baffone W. Palmieri G. F. (2018). Chitosan-based nanosystems and their exploited antimicrobial activity. Eur. J. Pharm. Sci. 117, 8–20. 10.1016/j.ejps.2018.01.046 29408419

[B95] Perteghella S. Rassu G. Gavini E. Obinu A. Bari E. Mandracchia D. (2021). Crocetin as new cross-linker for bioactive sericin nanoparticles. Pharmaceutics 13, 680. 10.3390/pharmaceutics13050680 34065101 PMC8150760

[B96] Qizilbash F. F. Ashhar M. U. Zafar A. Qamar Z. Baboota S. Ghoneim M. M. (2022). Thymoquinone-enriched naringenin-loaded nanostructured lipid carrier for brain delivery via nasal route: *in vitro* prospect and *in vivo* therapeutic efficacy for the treatment of depression. Pharmaceutics 14, 656. 10.3390/pharmaceutics14030656 35336030 PMC8953208

[B97] Rahimi A. Alimohammadi M. Faramarzi F. Alizadeh-Navaei R. Rafiei A. (2022). The effects of apigenin administration on the inhibition of inflammatory responses and oxidative stress in the lung injury models: a systematic review and meta-analysis of preclinical evidence. Inflammopharmacology 30, 1259–1276. 10.1007/s10787-022-00994-0 35661071

[B98] Rajitha P. Gopinath D. Biswas R. Sabitha M. Jayakumar R. (2016). Chitosan nanoparticles in drug therapy of infectious and inflammatory diseases. Expert Opin. Drug Deliv. 13, 1177–1194. 10.1080/17425247.2016.1178232 27087148

[B99] Rajput A. Pingale P. Dhapte-Pawar V. (2022). Nasal delivery of neurotherapeutics *via* nanocarriers: facets, aspects, and prospects. Front. Pharmacol. 13, 979682. 10.3389/fphar.2022.979682 36176429 PMC9513345

[B100] Ran D. Hong W. Yan W. Mengdie W. (2021). Properties and molecular mechanisms underlying geniposide-mediated therapeutic effects in chronic inflammatory diseases. J. Ethnopharmacol. 273, 113958. 10.1016/j.jep.2021.113958 33639206

[B101] Rashki S. Asgarpour K. Tarrahimofrad H. Hashemipour M. Ebrahimi M. S. Fathizadeh H. (2021). Chitosan-based nanoparticles against bacterial infections. Carbohydr. Polym. 251, 117108. 10.1016/j.carbpol.2020.117108 33142645

[B102] Rothmore J. (2020). Antidepressant-induced sexual dysfunction. Med. J. Aust. 212, 329–334. 10.5694/mja2.50522 32172535

[B103] Ruan S. Li J. Ruan H. Xia Q. Hou X. Wang Z. (2024). Microneedle-mediated nose-to-brain drug delivery for improved Alzheimer's disease treatment. J. Control Release 366, 712–731. 10.1016/j.jconrel.2024.01.013 38219911

[B104] Sagit M. Polat H. Gurgen S. G. Berk E. Guler S. Yasar M. (2017). Effectiveness of quercetin in an experimental rat model of allergic rhinitis. Eur. Arch. Otorhinolaryngol. 274, 3087–3095. 10.1007/s00405-017-4602-z 28493194

[B105] Şahin A. Sakat M. S. KıLıç K. Aktan B. Yildirim S. Kandemir F. M. (2021). The protective effect of naringenin against ovalbumin-induced allergic rhinitis in rats. Eur. Arch. Otorhinolaryngol. 278, 4839–4846. 10.1007/s00405-021-06769-7 33772317

[B106] Sanidad K. Z. Sukamtoh E. Xiao H. Mcclements D. J. Zhang G. (2019). Curcumin: recent advances in the development of strategies to improve oral bioavailability. Annu. Rev. Food Sci. Technol. 10, 597–617. 10.1146/annurev-food-032818-121738 30633561

[B107] Seo E. J. Fischer N. Efferth T. (2018). Phytochemicals as inhibitors of NF-κB for treatment of Alzheimer's disease. Pharmacol. Res. 129, 262–273. 10.1016/j.phrs.2017.11.030 29179999

[B108] Shrewsbury S. B. (2023). The upper nasal space: option for systemic drug delivery, mucosal vaccines and nose-to-brain. Pharmaceutics 15, 1720. 10.3390/pharmaceutics15061720 37376168 PMC10303426

[B109] Sinha V. R. Singla A. K. Wadhawan S. Kaushik R. Kumria R. Bansal K. (2004). Chitosan microspheres as a potential carrier for drugs. Int. J. Pharm. 274, 1–33. 10.1016/j.ijpharm.2003.12.026 15072779

[B110] Small P. Keith P. K. Kim H. (2018). Allergic rhinitis. Allergy Asthma Clin. Immunol. 14, 51. 10.1186/s13223-018-0280-7 30263033 PMC6156899

[B111] Soane R. Frier M. Perkins A. Jones N. Davis S. Illum L. (1999). Evaluation of the clearance characteristics of bioadhesive systems in humans. Int. J. Pharm. 178, 55–65. 10.1016/s0378-5173(98)00367-6 10205625

[B112] Sun M. Yu X. Wang T. Bi S. Liu Y. Chen X. (2019). Nasal adaptive chitosan-based nano-vehicles for anti-allergic drug delivery. Int. J. Biol. Macromol. 135, 1182–1192. 10.1016/j.ijbiomac.2019.05.188 31154036

[B113] Sun C. Zheng W. Wang L. Du Q. (2023). Gastrodin prevents neuronal apoptosis and improves neurological deficits in traumatic brain injury rats through PKA/CREB/Bcl2 axis. Front. Biosci. Landmark Ed. 28, 93. 10.31083/j.fbl2805093 37258463

[B114] Tai J. Lee K. Kim T. H. (2021). Current perspective on nasal delivery systems for chronic rhinosinusitis. Pharmaceutics 13, 246. 10.3390/pharmaceutics13020246 33578812 PMC7916625

[B115] Teng Z. Meng L. Y. Yang J. K. He Z. Chen X. G. Liu Y. (2022). Bridging nanoplatform and vaccine delivery, a landscape of strategy to enhance nasal immunity. J. Control Release 351, 456–475. 10.1016/j.jconrel.2022.09.044 36174803

[B116] Tong Z. Jie X. Chen Z. Deng M. Li X. Zhang Z. (2024). Borneol and lactoferrin dual-modified crocetin-loaded nanoliposomes enhance neuroprotection in HT22 cells and brain targeting in mice. Eur. J. Med. Chem. 276, 116674. 10.1016/j.ejmech.2024.116674 39004017

[B117] Tuo Q. Z. Zhang S. T. Lei P. (2022). Mechanisms of neuronal cell death in ischemic stroke and their therapeutic implications. Med. Res. Rev. 42, 259–305. 10.1002/med.21817 33957000

[B118] Walia V. Chaudhary S. K. Kumar Sethiya N. (2021). Therapeutic potential of mangiferin in the treatment of various neuropsychiatric and neurodegenerative disorders. Neurochem. Int. 143, 104939. 10.1016/j.neuint.2020.104939 33346032

[B119] Wang Y. Jiang S. Wang H. Bie H. (2017). A mucoadhesive, thermoreversible *in situ* nasal gel of geniposide for neurodegenerative diseases. PLoS One 12, e0189478. 10.1371/journal.pone.0189478 29240797 PMC5730156

[B120] Wang N. Wang H. Li L. Li Y. Zhang R. (2019). β-Asarone inhibits Amyloid-β by promoting autophagy in a cell model of Alzheimer's disease. Front. Pharmacol. 10, 1529. 10.3389/fphar.2019.01529 32009952 PMC6979317

[B121] Wang Q. S. Yan K. Li K. D. Gao L. N. Wang X. Liu H. (2021). Targeting hippocampal phospholipid and tryptophan metabolism for antidepressant-like effects of albiflorin. Phytomedicine 92, 153735. 10.1016/j.phymed.2021.153735 34601221

[B122] Wang X. Fu Y. Botchway B. O. A. Zhang Y. Zhang Y. Jin T. (2022). Quercetin can improve spinal cord injury by regulating the mTOR signaling pathway. Front. Neurol. 13, 905640. 10.3389/fneur.2022.905640 35669881 PMC9163835

[B123] Wang W. Wang Y. Wang F. Xie G. Liu S. Li Z. (2024a). Gastrodin regulates the TLR4/TRAF6/NF-κB pathway to reduce neuroinflammation and microglial activation in an AD model. Phytomedicine 128, 155518. 10.1016/j.phymed.2024.155518 38552431

[B124] Wang Y. Bai M. Wang X. Peng Z. Cai C. XI J. (2024b). Gastrodin: a comprehensive pharmacological review. Naunyn Schmiedeb. Arch. Pharmacol. 397, 3781–3802. 10.1007/s00210-023-02920-9 38165423

[B125] Wani A. AL Rihani S. B. Sharma A. Weadick B. Govindarajan R. Khan S. U. (2021). Crocetin promotes clearance of amyloid-β by inducing autophagy *via* the STK11/LKB1-mediated AMPK pathway. Autophagy 17, 3813–3832. 10.1080/15548627.2021.1872187 33404280 PMC8632093

[B126] Wei Q. Zhang J. Hong G. Chen Z. Deng W. He W. (2016). Icariin promotes osteogenic differentiation of rat bone marrow stromal cells by activating the ERα-Wnt/β-catenin signaling pathway. Biomed. Pharmacother. 84, 931–939. 10.1016/j.biopha.2016.09.107 27764755

[B127] Wong K. H. Riaz M. K. Xie Y. Zhang X. Liu Q. Chen H. (2019). Review of current strategies for delivering Alzheimer's disease drugs across the blood-brain barrier. Int. J. Mol. Sci. 20, 381. 10.3390/ijms20020381 30658419 PMC6358942

[B128] Wu E. L. Harris W. C. Babcock C. M. Alexander B. H. Riley C. A. Mccoul E. D. (2019). Epistaxis risk associated with intranasal corticosteroid sprays: a systematic review and meta-analysis. Otolaryngol. Head. Neck Surg. 161, 18–27. 10.1177/0194599819832277 30779679

[B129] Wu H. Li Y. Zhang Q. Wang H. Xiu W. Xu P. (2023). Crocetin antagonizes parthanatos in ischemic stroke *via* inhibiting NOX2 and preserving mitochondrial hexokinase-I. Cell Death Dis. 14, 50. 10.1038/s41419-023-05581-x 36681688 PMC9867762

[B130] Xiang Y. Long Y. Yang Q. Zheng C. Cui M. Ci Z. (2020). Pharmacokinetics, pharmacodynamics and toxicity of Baicalin liposome on cerebral ischemia reperfusion injury rats via intranasal administration. Brain Res. 1726, 146503. 10.1016/j.brainres.2019.146503 31605698

[B131] Xiao X. Xu X. Li F. Xie G. Zhang T. (2019). Anti-inflammatory treatment with β-asarone improves impairments in social interaction and cognition in MK-801 treated mice. Brain Res. Bull. 150, 150–159. 10.1016/j.brainresbull.2019.05.017 31129169

[B132] Xu D. Lu Y. R. Kou N. Hu M. J. Wang Q. S. Cui Y. L. (2020). Intranasal delivery of icariin via a nanogel-thermoresponsive hydrogel compound system to improve its antidepressant-like activity. Int. J. Pharm. 586, 119550. 10.1016/j.ijpharm.2020.119550 32554031

[B133] Xu D. Qiao T. Wang Y. Wang Q. S. Cui Y. L. (2021). Alginate nanogels-based thermosensitive hydrogel to improve antidepressant-like effects of albiflorin via intranasal delivery. Drug Deliv. 28, 2137–2149. 10.1080/10717544.2021.1986604 34617853 PMC8510626

[B134] Yang H. Li Q. Li L. Chen S. Zhao Y. Hu Y. (2022). Gastrodin modified polyurethane conduit promotes nerve repair via optimizing schwann cells function. Bioact. Mater 8, 355–367. 10.1016/j.bioactmat.2021.06.020 34541406 PMC8427216

[B135] Yao Y. Y. Ling E. A. Lu D. (2020). Microglia mediated neuroinflammation - signaling regulation and therapeutic considerations with special reference to some natural compounds. Histol. Histopathol. 35, 1229–1250. 10.14670/hh-18-239 32662061

[B136] Yu P. Wang L. Tang F. Zeng L. Zhou L. Song X. (2017). Resveratrol pretreatment decreases ischemic injury and improves neurological function Via sonic hedgehog signaling after stroke in rats. Mol. Neurobiol. 54, 212–226. 10.1007/s12035-015-9639-7 26738852

[B137] Yu P. Wang L. Tang F. Guo S. Liao H. Fan C. (2021). Resveratrol-mediated neurorestoration after cerebral ischemic injury - sonic hedgehog signaling pathway. Life Sci. 280, 119715. 10.1016/j.lfs.2021.119715 34116113

[B138] Yu S. Li D. Shi A. Long Y. Deng J. Ma Y. (2023). Multidrug-loaded liposomes prevent ischemic stroke through intranasal administration. Biomed. Pharmacother. 162, 114542. 10.1016/j.biopha.2023.114542 36989725

[B139] Zakaria F. H. Samhani I. Mustafa M. Z. Shafin N. (2022). Pathophysiology of depression: stingless bee honey promising as an antidepressant. Molecules 27, 5091. 10.3390/molecules27165091 36014336 PMC9416360

[B140] Zhang H. Lai Q. Li Y. Liu Y. Yang M. (2017a). Learning and memory improvement and neuroprotection of Gardenia jasminoides (fructus gardenia) extract on ischemic brain injury rats. J. Ethnopharmacol. 196, 225–235. 10.1016/j.jep.2016.11.042 27940085

[B141] Zhang Q. L. Fu B. M. Zhang Z. J. (2017b). Borneol, a novel agent that improves central nervous system drug delivery by enhancing blood-brain barrier permeability. Drug Deliv. 24, 1037–1044. 10.1080/10717544.2017.1346002 28687052 PMC8241164

[B142] Zhang J. Wang Y. Dong X. Liu J. (2018). Crocetin attenuates inflammation and amyloid-β accumulation in APPsw transgenic mice. Immun. Ageing 15, 24. 10.1186/s12979-018-0132-9 30450117 PMC6208089

[B143] Zhang Y. L. Yu P. C. Liu P. (2019a). Using high-throughput metabolomics to discover perturbed metabolic pathways and biomarkers of allergic rhinitis as potential targets to reveal the effects and mechanism of geniposide. RSC Adv. 9, 17490–17500. 10.1039/c9ra02166c 35519866 PMC9064603

[B144] Zhang Z. Wang X. Zhang D. Liu Y. Li L. (2019b). Geniposide-mediated protection against amyloid deposition and behavioral impairment correlates with downregulation of mTOR signaling and enhanced autophagy in a mouse model of alzheimer's disease. Aging (Albany NY) 11, 536–548. 10.18632/aging.101759 30684442 PMC6366989

[B145] Zhang Y. Zhao Y. Dong D. Li X. Li Z. Li S. (2020). Effects of isosorbide mononitrate loaded nanoparticles conjugated with anti-staphylococcus aureus α-toxin on *Staphylococcus aureus* biofilms. Exp. Ther. Med. 19, 1267–1274. 10.3892/etm.2019.8344 32010298 PMC6966144

[B146] Zhang L. Yang S. Huang L. Ho P. C. (2020a). Poly (ethylene glycol)-block-poly (D, L-lactide) (PEG-PLA) micelles for brain delivery of baicalein through nasal route for potential treatment of neurodegenerative diseases due to oxidative stress and inflammation: an *in vitro* and *in vivo* study. Int. J. Pharm. 591, 119981. 10.1016/j.ijpharm.2020.119981 33069896

[B147] Zhang W. Tang R. Ba G. Li M. Lin H. (2020b). Anti-allergic and anti-inflammatory effects of resveratrol *via* inhibiting TXNIP-oxidative stress pathway in a mouse model of allergic rhinitis. World Allergy Organ J. 13, 100473. 10.1016/j.waojou.2020.100473 33133334 PMC7586246

[B148] Zhang Z. Gao W. Wang X. Zhang D. Liu Y. Li L. (2020c). Geniposide effectively reverses cognitive impairment and inhibits pathological cerebral damage by regulating the mTOR signal pathway in APP∕PS1 mice. Neurosci. Lett. 720, 134749. 10.1016/j.neulet.2020.134749 31935433

[B149] Zhang W. Zhang F. Hu Q. Xiao X. Ou L. Chen Y. (2021). The emerging possibility of the use of geniposide in the treatment of cerebral diseases: a review. Chin. Med. 16, 86. 10.1186/s13020-021-00486-3 34454545 PMC8400848

[B150] Zhao C. Lv C. Li H. Du S. Liu X. Li Z. (2016). Geniposide protects primary cortical neurons against oligomeric Aβ1-42-Induced neurotoxicity through a mitochondrial pathway. PLoS One 11, e0152551. 10.1371/journal.pone.0152551 27046221 PMC4821580

[B151] Zhou Y. J. Wang H. Sui H. H. Li L. Zhou C. L. Huang J. J. (2016). Inhibitory effect of baicalin on allergic response in ovalbumin-induced allergic rhinitis Guinea pigs and lipopolysaccharide-stimulated human mast cells. Inflamm. Res. 65, 603–612. 10.1007/s00011-016-0943-0 27043920

[B152] Zhou Z. Lu J. Liu W. W. Manaenko A. Hou X. Mei Q. (2018). Advances in stroke pharmacology. Pharmacol. Ther. 191, 23–42. 10.1016/j.pharmthera.2018.05.012 29807056

[B153] Zhu T. Meng X. B. Dong D. X. Zhao L. Y. Qu M. W. Sun G. B. (2021a). Xuesaitong injection (lyophilized) combined with aspirin and clopidogrel protect against focal cerebral ischemic/reperfusion injury in rats by suppressing oxidative stress and inflammation and regulating the NOX2/IL-6/STAT3 pathway. Ann. Palliat. Med. 10, 1650–1667. 10.21037/apm-20-1681 33222458

[B154] Zhu T. Wang L. Xie W. Meng X. Feng Y. Sun G. (2021b). Notoginsenoside R1 improves cerebral ischemia/reperfusion injury by promoting neurogenesis *via* the BDNF/Akt/CREB pathway. Front. Pharmacol. 12, 615998. 10.3389/fphar.2021.615998 34025400 PMC8138209

[B155] Zhu T. Wang L. Wang L. P. Wan Q. (2022). Therapeutic targets of neuroprotection and neurorestoration in ischemic stroke: applications for natural compounds from medicinal herbs. Biomed. Pharmacother. 148, 112719. 10.1016/j.biopha.2022.112719 35168073

